# The Histone Deacetylase Family: Structural Features and Application of Combined Computational Methods

**DOI:** 10.3390/ph17050620

**Published:** 2024-05-10

**Authors:** Antonio Curcio, Roberta Rocca, Stefano Alcaro, Anna Artese

**Affiliations:** 1 Dipartimento di Scienze della Salute, Campus “S. Venuta”, Università degli Studi “Magna Græcia” di Catanzaro, Viale Europa, 88100 Catanzaro, Italy; antonio.curcio@unicz.it (A.C.); alcaro@unicz.it (S.A.); artese@unicz.it (A.A.); 2 Net4Science S.r.l., Università degli Studi “Magna Græcia” di Catanzaro, Viale Europa, 88100 Catanzaro, Italy

**Keywords:** histone deacetylases (HDACs), HDACs structure, HDACs inhibitors, molecular modeling, drug design

## Abstract

Histone deacetylases (HDACs) are crucial in gene transcription, removing acetyl groups from histones. They also influence the deacetylation of non-histone proteins, contributing to the regulation of various biological processes. Thus, HDACs play pivotal roles in various diseases, including cancer, neurodegenerative disorders, and inflammatory conditions, highlighting their potential as therapeutic targets. This paper reviews the structure and function of the four classes of human HDACs. While four HDAC inhibitors are currently available for treating hematological malignancies, numerous others are undergoing clinical trials. However, their non-selective toxicity necessitates ongoing research into safer and more efficient class-selective or isoform-selective inhibitors. Computational techniques have greatly facilitated the discovery of HDAC inhibitors that achieve the desired potency and selectivity. These techniques encompass ligand-based strategies such as scaffold hopping, pharmacophore modeling, three-dimensional quantitative structure–activity relationships (3D-QSAR), and structure-based virtual screening (molecular docking). Additionally, advancements in molecular dynamics simulations, along with Poisson–Boltzmann/molecular mechanics generalized Born surface area (PB/MM-GBSA) methods, have enhanced the accuracy of predicting ligand binding affinity. In this review, we delve into the ways in which these methods have contributed to designing and identifying HDAC inhibitors.

## 1. Introduction

Histone deacetylases (HDACs), also known as lysine deacetylases (KDACs), are proteolytic enzymes that depend on either zinc (Zn^2+^) or nicotinamide adenine dinucleotide (NAD^+^). They play a crucial role in transcriptional repression and chromatin condensation by regulating the acetylation state of lysine residues on histone tails [1]. HDACs achieve this by removing the acetate moiety from acetylated ε-amino groups on histone lysines and other non-histone proteins, thus regulating and modulating several pivotal biological signaling pathways [2]. After deacetylation, the positively charged N-terminal residues of amino acids interact with DNA phosphate groups, leading to the repression of gene transcription. By contrast, acetyltransferases (HATs) carry out the acetylation of histone lysines, thus neutralizing the positive charge of the lysine residue and relaxing the chromatin structure [3].

Furthermore, HDACs can indirectly influence other post-translational modifications (PTMs) by removing the acetyl group from lysine residues, thereby allowing other changes, such as ubiquitination, to occur at those sites [4]. 

The enzymatic activities of HDAC isoforms are crucial for maintaining various normal physiological processes, including cell proliferation, apoptosis, neurogenesis, and epigenetic regulation. An imbalance between histone acetylation and deacetylation can lead to a variety of diseases, including neurodegenerative and cardiovascular disorders, autoimmune diseases, metabolic disorders, diabetes, and cancer [5,6].

Due to their contributions in these pathophysiological conditions, HDACs have become attractive and significant targets, especially in cancer research [7,8]. Specifically, abnormal HDAC expression and deacetylation activity have become a major focus in the study of cancer prevention and progression [9,10].

Contemporary advancements in computational methods for designing HDAC inhibitors highlight innovative and multifaceted screening and design strategies. These approaches markedly improve the quality of identified compounds as potential HDAC inhibitors. Specifically, the use of multilayered computational methods reduces the risk of false positive hits, enhancing the ability to select specific inhibitors through diverse filtering and scoring functions. Thus, this review aims to investigate how the integration of diverse approaches and methodologies has substantially enhanced the reliability of compounds identified in the domain of HDAC inhibitor discovery.

### 1.1. Classification of HDAC Family

**Figure 1:**
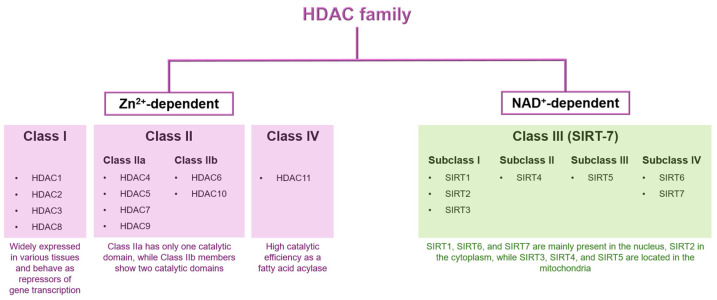
Schematic classification of HDAC family.

As reported in the recent review of Han and co-workers [11], so far, according to their 3D structure, function, and sequence homology, 18 isoforms of human HDACs have been identified. Based on their intracellular localization and tissue distribution specificity, they can be divided into four subfamilies (Class I, Class II, Class III, and Class IV) characterized by different biological functions (Figure 1) [12]. 

Class I subfamily includes HDAC1, HDAC2, HDAC3, and HDAC8. These isoforms are widely expressed in various tissues [13] and behave as repressors of gene transcription. HDAC1 and HDAC2 are highly homologous and are involved in cell proliferation, cell cycle regulation, and apoptosis [14]. HDAC3 plays a crucial role in cell cycle and DNA damage response [15], while HDAC8 is responsible for smooth muscle cell differentiation [16]. The Class II subfamily, which includes HDAC4, HDAC5, HDAC6, HDAC7, HDAC9, and HDAC10, can be further divided into two groups: Class IIa (HDAC4, HDAC5, HDAC7, and HDAC9) and Class IIb (HDAC6 and HDAC10) [17]. Class IIa HDACs possess a single catalytic domain, whereas Class IIb members have two catalytic domains [18]. HDAC4 and HDAC5, belonging to Class IIa, are found in the brain, heart, and skeletal muscle [19]. In contrast, HDAC7 is mainly expressed in the heart, lung, placenta, pancreas, skeletal muscle, and thymus [20]. HDAC9 is mainly expressed in the brain and skeletal muscle [21]. On the contrary, HDAC6 and HDAC10, which are representative of Class IIb, are expressed in the heart, skeletal muscle and brain [22], and in the liver, spleen, and kidney, respectively [23]. HDAC11, the only Class IV enzyme, shows a high catalytic efficiency as a fatty acid acylase and is present in the brain, heart, kidney, testis, and skeletal muscle [24]. 

The Class III subfamily, known as Sirtuins, comprises seven members (SIRT1-7). Named for their resemblance to the yeast Sir2 protein, Sirtuins utilize NAD^+^ to carry out their ADP-ribosyltransferase and histone deacetylase activities [25]. The SIR2 regulator family is divided into four subclasses: I, II, III, and IV. Subclass I consists of SIRT1, SIRT2, and SIRT3 proteins; subclass II contains SIRT4 protein; subclass III includes SIRT5 protein; and subclass IV comprise SIRT6 and SIRT7 proteins [26]. SIRT1, SIRT6, and SIRT7 are mainly present in the nucleus, SIRT2 in the cytoplasm, while SIRT3, SIRT4, and SIRT5 are located in the mitochondria [27]. Classes I, II, and IV HDACs exert their catalytic activity by means of Zn^2+^ ions and show a high homology of their catalytic core structural domain [28,29], with more remarkable variations in the sequences and structures outside the catalytic domain. By contrast, Class III HDACs are totally different from other HDACs [30,31], since they are NAD+-dependent Sir2 super proteins and their deacetylase reaction does not need Zn^2+^ direct involvement [32].

### 1.2. HDAC Structure and Function

HDACs generally possess a core structural domain called the HDAC structural domain, which includes two highly conserved regions: the HDAC N-terminal and the HDAC central structural domains [6]. These domains feature critical catalytic sites, such as zinc ions and arginine residues, essential for their enzymatic activity [33]. In contrast, the HDAC C-terminal domain is more variable, with differences in length and amino acid sequences across different HDAC types [34]. Additionally, HDACs can interact with various proteins, including those involved in cell cycle regulation and transcription factors [35].

Among the earliest discovered members of the Class I HDAC family was Rpd3 from budding yeast, along with human HDACs 1, 2, 3, and 8. These proteins are categorized into Class I due to their shared structural characteristics. The N-terminal regions of these HDACs contain the catalytic domains, which are essential for their enzymatic activity. Notably, the sequences of these catalytic domains exhibit a substantial degree of conservation, ranging between 40% and 70%, when compared to the catalytic domain found in yeast Rpd3 [36]. Also, HDACs1-3 possess C-terminal extensions of varying lengths, which can be subject to phosphorylation, enhancing their deacetylase activity and influencing the formation of co-inhibitory complexes [37]. These three isozymes are found within large multiprotein complexes [38]. HDAC1 and HDAC2 are strictly confined to the nucleus. On the other hand, HDAC3 features both a nuclear localization signal (NLS) and a nuclear export signal (NES), which means its presence within the nucleus can be dynamic. The localization of HDAC3 is influenced by various factors, including the specific cell type and the surrounding environmental conditions [39]. However, all three isozymes collectively play a significant role as nuclear deacetylases. 

**Figure 2:**
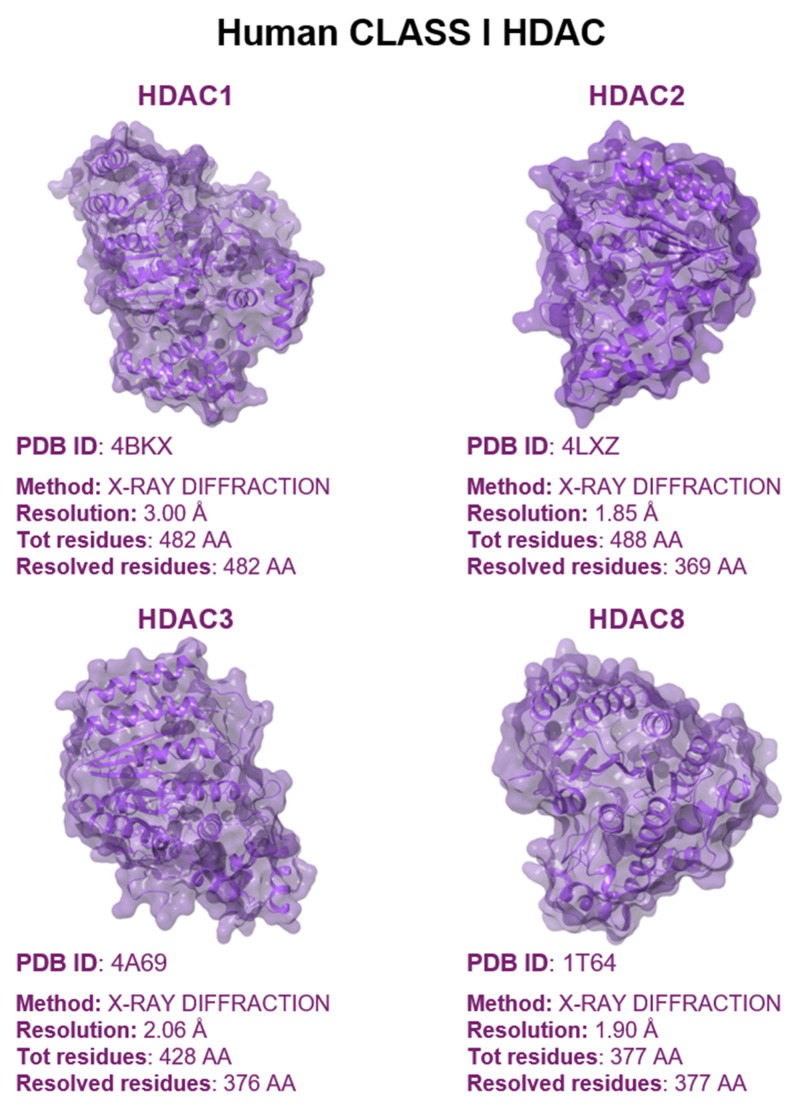
A 3D structure available for human Class I HDAC. HDAC1 in complex with the dimeric ELM2-SANT domain of MTA1 from the NuRD complex (PDB code 4BKX) [46]; HDAC2 in complex with SAHA (Vorinostat) (PDB code 4LXZ) [47]; HDAC3 bound to corepressor and inositol tetraphosphate (PDB code 4A69) [45]; HDAC8 complexed with Trichostatin A (PDB code 1T64) [45].

**Figure 3:**
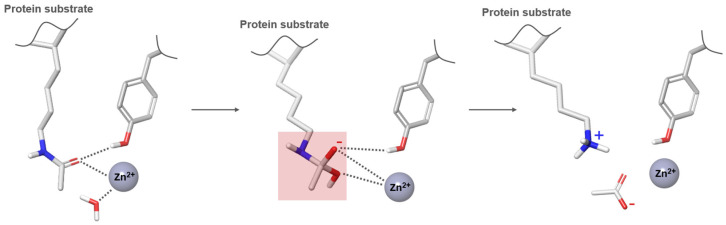
Dynamics and catalytic mechanisms of zinc ion-dependent HDACs during the deacylation process. The hydrolyzing portion is highlighted in red.

HDAC8 differentiates itself from other Class I isozymes through the absence of a C-terminal extension region. Notably, HDAC8 demonstrates significant histone deacetylase activity and substrate selectivity even in its isolated form, suggesting a relatively independent functional capacity [40]. Structural analysis through crystallography reveals that the catalytic domain of Class I HDACs comprises approximately 400 amino acid residues, sharing a common structural framework. The enzyme’s structure is anchored by a core that includes eight parallel β-strands organized into a β-sheet, which is flanked by over thirteen α-helices and extended loops expanding from the C-terminus of the β-sheet. This arrangement creates a narrow hydrophobic channel [4]. Within HDAC8, this channel is specifically outlined by residues such as Phe152, Phe208, His180, Gly151, Met274, and Tyr306. However, in other members of the Class I HDAC subfamily (HDAC1-3), most of these residues are conserved, with the exception of Met274, which is replaced by leucine residues [41]. These conserved hydrophobic residues characterize the binding sites for the substrate. In the catalytic mechanism, the acetylated lysine of the substrate docks into the catalytic core pocket at the bottom of the hydrophobic channel, interacting with the zinc ion bound therein. Under normal physiological conditions, the hydrophobic channel accommodates the acetylated lysine side chain, containing four methylene groups of the substrate. At the bottom of the channel, the Zn^2+^ ion forms a chelate complex with the His180, Asp178, and Asp267 of the HDAC, the oxygen atom from the substrate’s acetyl group, and a water molecule participating in the hydrolysis process. Besides the substrate binding site and the zinc ion binding site, the catalytic domain of the HDAC also features two additional metal ion binding sites, known as Site 1 and Site 2 [42]. Site 1 is located close to the zinc ion binding site, while Site 2 is on the periphery of the catalytic domain, near the N-terminal end of the β-fold bundle. The presence of two metal ions within the enzyme structure is crucial in promoting its stability. Additionally, the metal ion located at Site 1 may potentially serve a functional purpose in the deacetylase reaction. This suggests that the metal ions not only provide structural support, but also contribute to the enzyme’s catalytic activity, making them essential components of the enzyme’s overall functionality. The main function of Class I HDACs is to remove acetyl groups from histones within complexes, thereby catalyzing enzymatic reactions. These complexes play a crucial role in regulating HDAC activity and specificity, and are also subject to regulation by other transcription factors. By means of this regulation, the complexes are able to bind to chromosomes and precisely target specific temporal and spatial locations for deacetylation. Besides, phosphatidylinositol, a conserved regulator within the Class I HDAC co-inhibitor complex, has been found to enhance HDAC enzymatic activity. However, it has been highlighted that effective activation of HDAC enzymatic activity requires the presence of both co-inhibitors and phosphatidylinositol. The interactions of phosphatidylinositol within the substrate binding channel alter the conformation of the channel, facilitating substrate access to the catalytic active site [43]. The involvement of polyinositol in this complex also hints at a possible connection between epigenetics and cellular metabolism. It is worth noting that HDAC8, unlike other Class I isoenzymes, has a higher catalytic efficiency for acyl-lysine substrates than for acetyl-lysine. Furthermore, HDAC8 operates independently of multiprotein complexes. What is particularly interesting is that the three-dimensional structural model of human HDAC8 in the PDB database reveals that the secondary structure consists of eleven or thirteen α-helices and eight β-folds [44]. The analysis of the PDB database reveals several distinctive features of HDAC8 compared to HDACs1-3 (Figure 2). Specifically, in HDAC8, α-helix H1 assumes a standard α-helix conformation, whereas loop L1 is two amino acids shorter compared to HDAC3. Moreover, loop L6 in HDAC8 includes a proline residue, which positions the loop slightly farther from the catalytic site. This results in a more open catalytic pocket in HDAC8, potentially improving the substrate’s access to the catalytic core. These structural distinctions enhance the alignment of HDAC8’s catalytic core and facilitate more efficient substrate binding [45]. Understanding the crystal structure of HDAC8 is pivotal for gaining insights into the structural dynamics and catalytic processes of zinc-dependent HDACs, particularly those in the Class I subgroup (Figure 3) [45]. Given that HDAC8 shares substantial sequence identity with HDAC1 (40%), HDAC2 (41%), and HDAC3 (41%), and that the catalytic activity centers of zinc ion-dependent HDACs are conserved, the information obtained from HDAC8’s crystal structure can provide valuable insights into this particular group of enzymes.

**Figure 4:**
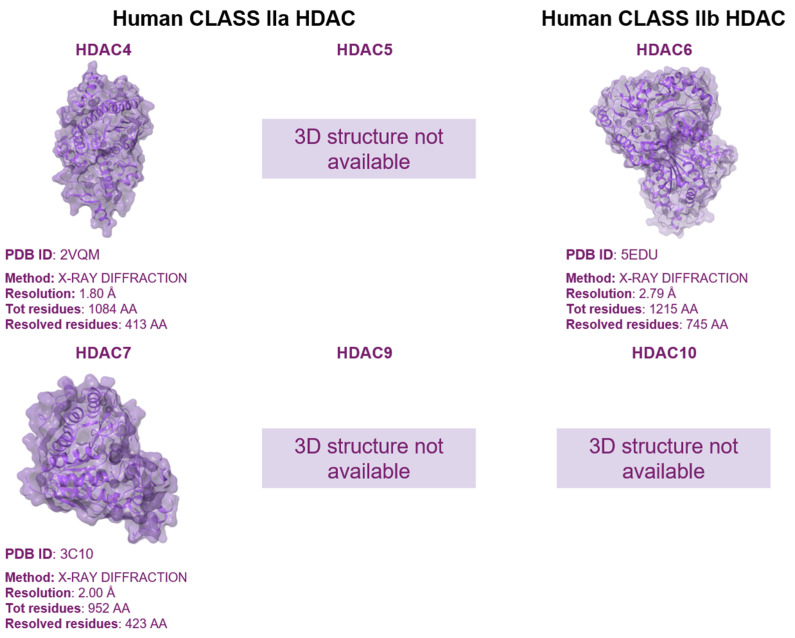
A 3D structure available for human Class IIa and IIb HDAC. HDAC4 catalytic domain bound to a hydroxamic acid inhibitor (PDB code 2VQM) [61]; HDAC6 with catalytic domain 2 in complex with Trichostatin A (PDB code 5EDU) [62]; HDAC7 catalytic domain in complex with Trichostatin A (PDB code 3C10) [63].

Class II enzymes are categorized into Type a and Type b. Type a HDACs include HDAC4, 5, 7, and 9, whereas Type b consists of the recently discovered HDAC6 and HDAC10 [48] (Figure 4). Class IIa HDACs share a common catalytic core domain at their C-terminus and feature a distinctive, conserved N-terminal extension with multiple binding sites. Specifically, this region includes the MEF2 (myogenic transcription factor 2) binding site, which is crucial for inhibiting muscle cell differentiation. Additionally, the region contains several phosphorylated serine sites that regulate enzyme localization and interactions with transcription factors and co-blockers [49]. Class IIa HDACs can move easily between the nucleus and cytoplasm. The catalytic domains of Class IIa HDACs and Class I HDACs share similarities, featuring an α/β structure with several loops that form the substrate binding channel and catalytic active site. The active site contains a single zinc ion, whereas two potassium/sodium ion binding sites are also present. While the histone deacetylation domain is conserved across Class IIa HDACs, the distinct catalytic activity of this class remains incompletely comprehended [50]. Unlike Class I and IIb HDACs, IIa HDACs feature a conserved histidine residue instead of a tyrosine residue at their active site, leading to limited deacetylation activity [51,52]. However, this constraint does not hinder their role as transcriptional repressors. In this regard, IIa HDACs contribute to epigenetic functions not only through deacetylation but also by recruiting Class I HDACs and interacting with transcription factors via their N-terminal binding site [53]. On the other hand, HDAC6, belonging to Class IIb, is mainly observed in the cytoplasm and comprises distinct structural domains such as NLS (localization signal region), NES1 and NES2 (leucine-rich nuclear export signal regions), DD1 and DD2 (tandem deacetylation catalytic regions), SE14 (serine–glutamate-containing tetradecapeptide repeat region), and ZnF-UBP (ubiquitin-binding zinc finger structure) [54]. Despite containing a nuclear localization signal, HDAC6’s cytoplasmic localization is primarily governed by NES and SE14, facilitating its translocation and anchoring in the cytoplasm [55,56]. 

The role of hadC6 within the cytoplasm remained unknown for a considerable period. However, in 2002, its characterization as a major histone deacetylase and its diverse array of non-histone substrates, including α-microtubulin, cortactin, Ku70, and HSP90, were elucidated [57]. Tubulin stands out as the principal substrate of HDAC6, influencing cytoskeletal dynamics, intracellular transport, and cellular motility. HDAC6 regulates microtubule assembly and the positioning of microtubule motor complexes, affecting cell motility and the interaction of cortical actin with microfilaments [58]. Therefore, the inhibition of HDAC6 leads to the hyperacetylation of microtubule proteins, which improves intracellular vesicular transport and is linked to neurological disorders such as Parkinson’s and Huntington’s disease [59]. Additionally, HDAC6 participates in crucial intracellular signaling pathways, underscoring its cellular significance. Conversely, HDAC10 operates as a transcriptional repressor, capable of shuttling between the nucleus and cytoplasm. It contains two conserved deacetylation catalytic domains at the N-terminal end. Although the C-terminal region resembles the N-terminal sequence, it does not possess deacetylase activity. The cytoplasmic localization is determined by a leucine-rich domain at the C-terminal end. The cytoplasmic localization is dictated by the leucine-rich domain situated at the C-terminal end [60]. HDAC10 exhibits interactions with HDAC3 akin to Type a HDACs, yet it possesses a distinct capability to function independently as a deacetylase.

HDAC11 is the only member of Class IV HDAC, which has fewer similarities to Class I and II HDACs. Among the identified HDACs, HDAC11 is the shortest, and is primarily comprised of the core catalytic domain that exhibits exclusive deacetylase activity [64]. This protein can be found in both the nucleus and cytoplasm of cells and has tissue-specific expression, with notable abundance in the kidney, heart, brain, skeletal muscle, testis, and other tissues [65]. In vivo, HDAC11 can also form complexes with HDAC6 [66]. Despite being the most recently discovered isoenzyme, HDAC11 remains one of the least studied and understood proteins within the HDAC family, with its 3D structure currently unavailable.

**Figure 5:**
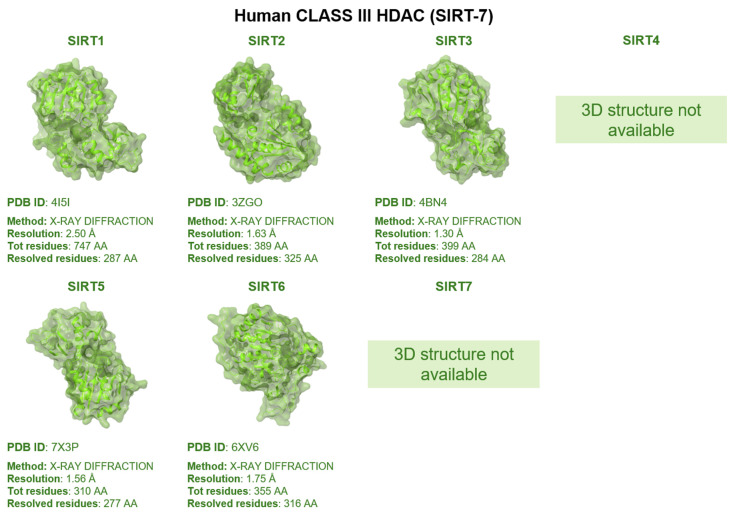
A 3D structure available for human Class III HDAC (SIRT-7). SIRT1 catalytic domain bound to NAD and an EX527 analog (PDB code 4I5I) [79]; SIRT2 apoform (PDB code 3ZGO) [80]; SIRT3 in complex with ADP-ribose (PDB code 4BN4) [81]; SIRT5 in complex with diazirine inhibitor 9 [PDB code 7X3P] [82]; SIRT6 in complex with ADP-ribose [83].

Class III HDACs are a group of NAD^+^-dependent deacetylases, which are capable of catalyzing the deacetylation of histone and non-histone substrates [67]. This class of HDACs belongs to the Sirtuin protein family, which has seven members in humans and are known as SIRT1–7. In yeast, this family is represented by the Sir2 protein [68]. For human Sirtuin proteins SIRT1, 2, 3, 5, and 6, the crystal structures of their catalytic core domains have been successfully resolved (Figure 5). These structures exhibit a well-organized comprehensive configuration, which can be ascribed to the selective evolutionary process and the preservation of the catalytically active region’s sequence. The catalytic domain of these proteins shows an elliptical configuration and is organized into two prominent and two smaller structural segments, each spanning approximately 270 amino acids. The more conserved of the two larger segments features a Rossmann fold architecture, which includes a central β-sheet surrounded by six β-strands. Additionally, this domain boasts several α-helices forming pockets for NAD^+^ accommodation and binding. On the other hand, the smaller domain is more variable and comprises two modules extending from the large structural domain, including a conserved Zn^2+^ binding element and a region of α-helices with relatively high variability [69]. The zinc finger domain consists of three β-strands arranged in a reverse parallel pattern along with a single α-helix [70]. The highly conserved four-loop region connecting the structural domains forms the substrate binding pocket crucial for catalytic activity. Of particular importance is the largest loop, known as the β1-α2 loop or cofactor binding loop. This loop, integral to the NAD^+^ binding site, exhibits significant structural flexibility, which is crucial for the enzyme’s catalytic activity.

Research has highlighted that SIRT1–3 display significant deacetylating activity, differing from the lower activity observed in SIRT5–7 [71]. According to several studies, different SIRTs may exhibit higher activity toward novel acylations. Notably, SIRT4 does not show any significant deacetylation activity. On the other hand, SIRT1–2 demonstrate significant activity against various acylations. This suggests that different SIRTs may have distinct functions and roles within cells, contributing to various biological processes [68]. Moreover, SIRT2 has the ability to facilitate the removal of benzoyl groups from histone lysine both in laboratory experiments and within living organisms [72]. SIRT5, a Sirtuin protein, offers an array of enzymatic activities, including debenzoylation, which can modify specific amino acid residues on target proteins. Furthermore, SIRT5 can act on different acyl-CoA derivatives, such as malonyl, butanoyl, and glutaryl groups, providing a wide range of regulatory functions. By contrast, SIRT4 and SIRT6 exhibit ADP ribosyltransferase activity, a post-translational modification that can alter protein function and localization. Notably, SIRT6 also exhibits debenzoylation activity on long-chain fatty acids, which can further diversify its functional range of activity [73]. SIRT7’s deacetylation activity is triggered by double-stranded DNA, resulting in histone H3 lysine 18 (H3K18) deacetylation within chromatin. Moreover, rRNA can enhance SIRT7’s long-chain fatty acylation activity, potentially exceeding its deacetylation activity [74]. The intracellular localization of the Sirtuin family deacetylases is clearly established. SIRT1, which shares close resemblance with yeast Sir2, has been the object of extensive research. SIRT3 is distributed in both the nucleus and mitochondria [75], while SIRT4 and SIRT5 are predominantly mitochondrial [76]. SIRT6 exclusively resides in the nucleus, whereas SIRT7 is specifically localized in the nucleolus [77]. In summary, the roles of SIRT1–7 in cells exhibit complexity and diversity [78].

HDACs are implicated in a range of diseases, spanning cancer, neurodegenerative disorders, and inflammatory conditions, underscoring their potential as therapeutic targets (Table 1) [84,85,86,87,88]. Particularly in cancer biology, their different functions are evident through their commonly altered expression across a variety of tumor types, emphasizing their significance as potential targets for cancer treatment (Table 2) [6,84,89,90,91]. 

As research into the structural complexities of HDACs advances, distinct differences among various subgroups and subtypes are being revealed. This understanding helps explain the selectivity of certain HDAC inhibitors. For instance, novel o-phenylenediamine HDAC inhibitors have been shown to specifically target HDAC1 and HDAC2 [92]. The analysis of HDAC1 and HDAC3 structures revealed a key difference: a substitution of Ser113 in HDAC1 with Tyr96 in HDAC3 at the bottom of the catalytic center. This variation likely contributes to the observed selectivity. However, most current HDAC inhibitors lack isoform selectivity due to the highly conserved amino acid sequence in the catalytic center of Zn^2+^-dependent HDACs [93]. On the other hand, the amino acid sequence surrounding the active site entrance on the protein surface remarkably differs among the isoforms. HDAC8, for example, has a shorter L1 loop near the active center entrance than other Class I members, resulting in a more expansive entrance and a more flexible protein surface. Researchers are employing an effective strategy to develop inhibitors that specifically target individual HDAC8 subtypes by considering the structural variations around the entrance to each subtype’s active site. A thorough grasp of the structural differences among HDAC isoforms will be crucial for developing potent and specific inhibitors (Table 3).

Interestingly, as extensively documented in the scientific literature, the functions of most HDAC isoforms undergo significant regulation through various post-translational modifications (PTMs), such as phosphorylations, acetylations, sumoylations and ubiquitinations. Additionally, HDACs can form complexes with other proteins (Table 3), thereby modulating their deacetylase activity [99]. In this context, multiple HDAC isoforms may participate in these complexes, where one isoform can influence the activity of another. For instance, research on human HDAC1 and HDAC2 has uncovered their coexistence in at least three distinct multi-protein complexes (Sin3, NuRD/NRD/Mi2 and CoREST), exerting mutual effects on their activities [100]. Furthermore, Fischle et al. demonstrated that the enzymatic activity of HDAC4, 5 and 7 relies on their association with the HDAC3/SMRT/N-CoR complex [101]. Specifically, the C-terminal zinc-binding domain of HDAC4 plays a critical role in substrate recognition and its association with the HDAC3–NCoR corepressor complex [102].

### 1.3. Mutation Effects on HDACs Biology

The dysregulation of HDACs, either through excessive or reduced activity, contributes to tumorigenesis by affecting apoptosis, differentiation and angiogenesis [6]. In particular, specific mutations can significantly alter the deacetylase activity of HDACs, thereby driving tumorigenesis and promoting cancer development [103]. Indeed, these mutations alter the dynamic regulation of histone acetylation and deacetylation processes catalyzed by lysine acetyltransferases (KATs) and HDACs [103]. Notably, mutations in HDACs, like HDAC2, HDAC4 and HDAC9, have been identified in various cancers (colon, breast, and prostate cancer) [104,105,106]. For instance, the recurrent frameshift mutation in exon1 of HDAC2 is particularly common in colon cancer and leads to a loss of measurable HDAC2 expression in mutant tumors [105]. This mutation confers resistance to HDAC inhibitors and alters gene expression to promote tumorigenesis [107]. In this regard, in vitro experiments demonstrated that HDAC2-deficient cells were unresponsive to HDAC inhibitors, such as Trichostatin A. These cells did not exhibit increased acetylation of histones H3 and H4, and their proliferation was not reduced compared to cells expressing wild-type HDAC2 [105]. On the other hand, mutations in other proteins, such as AT-Rich Interaction Domain 1A (ARID1A), can also influence HDAC activity and therapeutic responses [108,109]. ARID1A mutations are common in ovarian clear cell carcinomas (OCCCs) and endometrioid carcinomas (OECs), leading to loss of ARID1A protein expression and driving ovarian cancer progression [108,109]. Together, elevated HDAC2 expression is associated with poor outcomes in ovarian cancer [110]. Thus, given that EZH2 inhibition is synthetically lethal with ARID1A mutation, and the EZH2-containing PRC2 complex interacts with HDAC2, attempts were made to determine whether ARID1A regulates the interaction between EZH2 and HDAC2. Co-immunoprecipitation (coIP) analysis demonstrated an interaction between EZH2 and HDAC2 in ARID1A-mutated Ovarian Tumor-derived Cell Line 21G (TOV21G), and the restoration of wild-type ARID1A disrupted this interaction, suggesting that EZH2 did not interact with HDAC2 in ARID1A wild-type cells. Furthermore, ARID1A knockout amplified the growth inhibition caused by HDAC2 knockdown in ARID1A wild-type RMG1 cells while restoring wild-type ARID1A in ARID1A-mutated cells, reducing sensitivity to HDAC2 knockdown [111]. Notably, the observed growth inhibition induced by HDAC2 knockdown could be rescued by a short hairpin RNA (shRNA)-resistant wild-type HDAC2, but not by a catalytically inactive mutant HDAC2 H142A [112]. Given the involvement of the catalytic site, the sensitivity of HDAC2 to SAHA was evaluated in preclinical models of ARID1A-mutated ovarian cancers. ARID1A-mutated cells exhibited significantly lower half-maximal inhibitory concentration (IC50) of SAHA than ARID1A wild-type cells. Furthermore, SAHA treatment effectively inhibited the growth of xenografted ARID1A-mutated tumors and improved survival in mice bearing orthotopically transplanted ARID1A-mutated tumors, suggesting potential for achieving selectivity with pan-HDAC inhibitors in ARID1A-inactivated cells [111].

### 1.4. HDACs, HDACIs, Metabolism and Emerging Technologies like Omics

Histone acetylation and deacetylation processes are highly sensitive to changes in metabolite levels, which can impact the effectiveness of histone deacetylase inhibitors (HDACis) and the intrinsic HDAC activity. Additionally, HDACs have demonstrated regulatory effects on proteins beyond histones, including enzymes participating in metabolic pathways. There are several examples of metabolic pathways influenced by HDAC activity [113]. For instance, in several cancers, such as hepatocellular carcinoma (HCC), heightened aerobic glycolysis contributes to increased tumor growth, a phenomenon known as the Warburg effect [114]. The gluconeogenesis pathway suppresses aerobic glycolysis; hence, the inhibition of gluconeogenesis can further increase cancer cell growth. Yang et al. identified elevated levels of HDAC1 and HDAC2 in HCC tissues. HDAC1 and HDAC2 inhibit Fructose-1,6-bisphosphate (FBP1) expression, the key enzyme in the gluconeogenesis pathway, through histone H3K27 deacetylation at the FBP1 enhancer. This repression of gluconeogenesis promotes aerobic glycolysis and cancer progression. Knockdown of HDAC1 and HDAC2 resulted in increased FBP1 expression and reduced cell growth in HCC cell lines [115]. Thus, these deacetylases are not only implicated in epigenetic modifications, but also in metabolic or, for example, immune modulation [115]. Therefore, understanding the impact of HDACs on cancers through metabolic or other processes can reveal new potential targets [113]. Consequently, computational -omics techniques encompassing genomics, transcriptomics, proteomics, metabolomics and epigenomics can play crucial roles in elucidating the HDACs functionality and the inhibitory activity of HDACis, especially in the context of specific mutations [113]. For instance, transcriptomics can identify gene expression changes upon HDAC inhibition, while metabolomics can reveal metabolic alterations influenced by HDAC activity. Moreover, epigenomics techniques, like Chromatin Immunoprecipitation Sequencing (ChIP-seq), can elucidate the genomic regions targeted by HDACs [116]. 

Various -omics approaches have begun to elucidate the underlying mechanisms of therapeutic or toxic effects associated with HDACis. These inhibitors modulate the expression of genes involved in diverse biological pathways, including cell cycle regulation, cell death, metabolism, and stress responses in cancer cells [116]. Beyond epigenomic and transcriptomic profiling, recent advancements in proteomics, metabolomics and chemoproteomics have provided datasets relevant to HDACis (Table 4) [116]. 

For example, Zhu et al. conducted multi-omics analyses involving bulk RNA sequencing (RNA-seq), transposase accessible chromatin sequencing (ATAC-seq), and H3K27ac-targeted cleavage under targets and tagmentation sequencing (CUT&Tag-seq) in HDACi-treated CAR-T cells, revealing comprehensive epigenetic remodeling and functional alterations, including changes in chromatin accessibility, transcription factor interaction networks and regulation of T cell differentiation. Also, specific HDAC inhibitors, like M344 and Chidamide (selective class I inhibitors), notably suppressed HDAC1 expression in CD19-28ζ CAR-T cells [117]. Regarding metabolism, inhibition of HDAC activity by HDACis can affect various metabolic processes. Amoedo et al. demonstrated that sodium butyrate (NaB) and Trichostatin A (TSA) treatment of lung cancer cells led to increased oxygen consumption coupled with ATP synthesis, activation of the pentose phosphate pathway (PPP), and enhanced mitochondria-bound hexokinase activity, promoting glycolysis [118]. Also, a study conducted by Wardell et al. examined the impact of HDACis valproate (VPA) and suberoylanilide hydroxamic acid (SAHA) on metabolism within the context of multiple myeloma. These HDACis elicited various metabolic changes in the cells, including reduced levels of acetyl-CoA, a decreased expression of glucose transporter type 1 (GLUT1) and the inhibition of hexokinase 1 (HXK1) activity [118]. The latter two effects are associated with diminished glucose uptake and glycolysis, processes essential for energy production in cancer cells [119]. On the other hand, some metabolites can regulate HDAC and HDACi activity. For instance, trapoxin (TPX) acts as an irreversible inhibitor of HDAC1 and HDAC4 by covalently binding to these targets [120,121]. Additionally, metabolism can enhance the activity of specific HDACis, such as the reduction of the disulfide bond in depsipeptide to create an active compound like romidepsin, which exhibits potent anticancer effects in leukemias and lymphomas [122,123]. In summary, employing systems technologies like proteomics and metabolomics is instrumental in fully understanding the mechanism of action of HDAC inhibitors and evaluating their therapeutic activity [113].

## 2. HDAC Inhibitors

**Figure 6:**
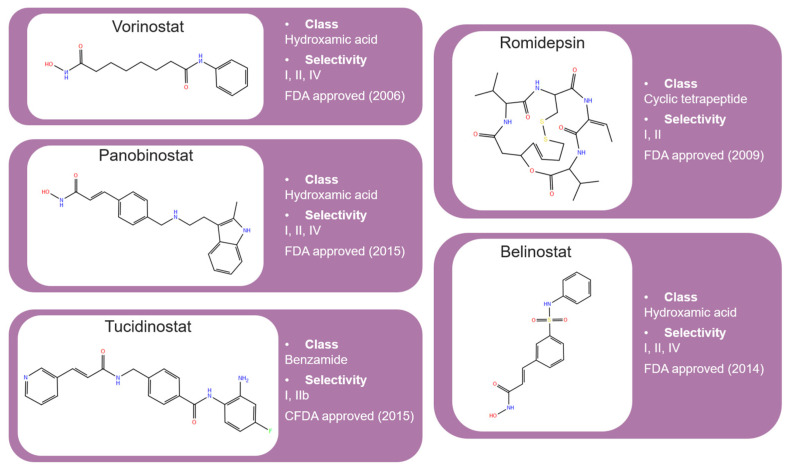
A 2D structure of the four HDACis (Vorinostat, Panobinostat, Romidepsin and Belinostat) approved by FDA for the treatment of various cancers and of Tucidinostat, the only HDACi approved by CFDA.

HDAC inhibitors (HDACis) are a class of pharmacological agents that have shown great potential in the treatment of cancer. These compounds primarily function by altering the acetylation status of histones, which play a crucial role in regulating gene expression. By modifying histone acetylation, HDACis can induce changes in chromatin structure, leading to the activation of silenced genes and the inhibition of genes that promote cell proliferation. HDACis have been shown to exert their anticancer effects through a variety of mechanisms, including apoptosis, cell cycle arrest and autophagy [124,125]. Clinical trials have primarily focused on developing targeted Class I/II HDAC inhibitors, such as isohydroxamic acids like SAHA and cyclic peptides. These inhibitors have shown the most promising activity in inhibiting HDACs. However, they face several challenges, including poor bioavailability, rapid metabolic breakdown, irreversible differentiation effects, and insufficient selectivity for cancer cells. To address these issues, it is essential to investigate the diverse functions of different HDACs in order to create more effective and targeted HDAC inhibitors. Additionally, HDAC inhibitors have exhibited effects on various cells and genes, indicating multiple antitumor mechanisms [126], including apoptosis and autophagy induction [127], tumor cell cycle arrest [128], and the inhibition of tumor cell angiogenesis [127]. In many cancer cells, HDAC inhibitors can induce cell death by activating either the extrinsic pathway, which affects receptor-mediated apoptosis, or the intrinsic pathway, which involves mitochondrial signaling [129]. Over the past few years, many HDAC inhibitors have been created either synthetically or by extracting them from natural sources [130]. The first natural hydroxamic acid known to inhibit HDACs was Trichostatin A (TSA). Vorinostat (suberoylanilide hydroxamic acid, SAHA), structurally similar to TSA, was the first FDA-approved HDAC inhibitor for the treatment of refractory cutaneous T-cell lymphoma (CTCL) [131]. At present, the US Food and Drug Administration (FDA) has given its approval to four HDACis for the treatment of various hematologic tumors as well as certain solid tumors (Figure 6) [132,133]. Moreover, Tucidinostat was approved in 2015 by the China Food and Drug Administration (CFDA) in the treatment of certain cancers [134].

**Figure 7:**
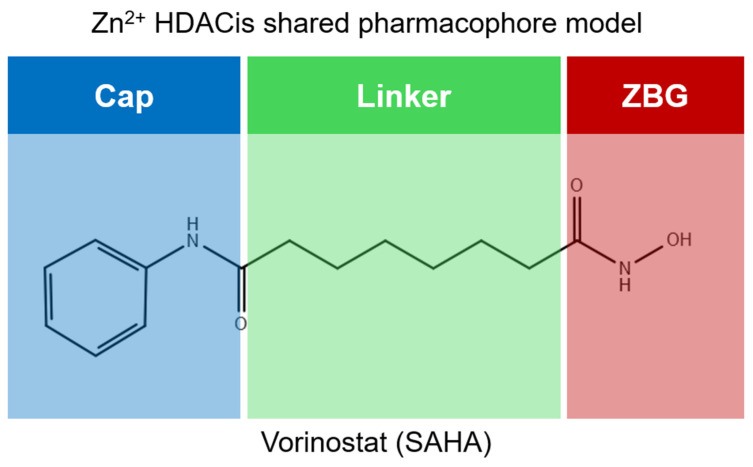
A 2D structure of Vorinostat showing the HDACis shared pharmacophore model.

Over the past two decades, there has been a significant expansion in the compound library of Zn^2+^-dependent HDAC inhibitors (HDACis). Despite the various structures of these compounds, whether synthesized or naturally occurring, a shared pharmacophore model predominates among most Zn^2+^ HDACis (Figure 7). This model comprises three essential elements:

(1) the cap structure (Surface Recognition Domain): this component generally features a hydrophobic aromatic group that interacts with the enzyme surface;
(2) Zn^2+^-binding group (ZBG): this group, which can include compounds such as isohydroxamic acid, carboxylic acid, or benzamide, binds to the Zn^2+^ ion at the enzyme’s catalytic site;
(3) linker: this element, which can be a linear chain (either saturated or unsaturated) or a hydrophobic long chain with a ring structure, connects the cap structure to the ZBG [135,136].

Studies of co-crystalized complexes between isohydroxamic acid HDAC inhibitors and HDACs have clarified how the cap structure interacts with amino acids near the enzyme’s catalytic site. Additionally, the zinc-binding group (ZBG) structure binds to the metal ion at the bottom of the active site, forming a stable complex [47]. The linker is a crucial component that plays a significant role in positioning the ZBG group within the active region of HDACs. The optimal length of the linker is of utmost importance, as it helps the ZBG group to chelate with Zn^2+^ and to establish hydrogen bonds with amino acids like histidylic acid and tyrosine. The extended linker chain engages with residues within the active site through van der Waals interactions, while the cap structure acts as a barrier, blocking access to the enzyme’s active site entrance [47]. HDACis work by competing with acetyl-lysine residues for binding to the enzyme’s active site, thereby blocking their interaction and disrupting the enzyme’s activity. However, alterations in any of the three components of the HDACis pharmacophore can influence their activity or selectivity, which can affect their therapeutic efficacy and safety. Isohydroxamic acid, benzamide, carboxylic acid, sulfhydryl groups, ketones, and epoxides are typical groups for ZBG. A comparative analysis of clinically used HDACis, including SAHA, entinostat (MS275), and valproic acid—each with distinct chelating groups—reveals notable differences in their effectiveness against HDACs. Notably, isohydroxamic acid exhibits the most potent zinc ion chelation capability [137]. Linker structures can differ in terms of their structural characteristics, such as lengths, saturation, unsaturation, linearity, cyclicality, and modifications. Scientists have found that modifying the linker can significantly affect the activity of an HDACi, making it more or less effective. Some of the most commonly used types of linkers include aliphatic chains, aromatic rings and vinyl–aromatic rings [138]. Docking and energy-optimized pharmacophore localization studies have revealed that a higher affinity for the target can be obtained with inhibitors containing at least one aromatic ring in their linker region. Moreover, the highest level of enzyme inhibition was achieved when the linker region (n) contained six carbon atoms. Phenyl, naphthyl and thiophene groups in the cap groups enhance the hydrophobic and high capacity of compounds, leading to improved HDAC inhibition. Additionally, the presence of substituents with higher lipophilicity, such as trifluoromethyl, tend to result in stronger HDAC inhibition. The effect is even more pronounced when methoxy and trifluoromethyl substitutions occur in the cap group at the adjacent, inter-, and *para*-positions. Lipophilicity unequivocally amplifies the hydrophobic interaction between the HDAC active site and its inhibitor, resulting in a marked increase in the inhibitor activity [139]. It is important to improve our understanding of the HDACi inhibition mechanism to develop drug compounds more effectively. One way to achieve this is by combining the pharmacophore model of HDAC inhibitors with structural insights into the enzymatic active region. This widely accepted approach enables the rational design and optimization of HDAC inhibitors by modifying their structure based on the three components of the pharmacophore.

The ZBG plays a pivotal role in the inhibitory activity of HDACis by binding to Zn^2+^ and its adjacent residues [48]. Using the type of ZBG to classify the six HDACIs, three primary categories are obtained (Table 5):

(1) Isohydroxamic acids, which encompass SAHA, Belinostat (PXD101), and Panobinostat (LBH589);
(2) Benzamide derivatives, exemplified by Mocetinostat (MGCD0103) and Chidamide;
(3) Cyclic peptides, represented by romidepsin (FK228) [140].

Moreover, there are multiple HDACi drugs currently being studied in preclinical and clinical trials [141].

### 2.1. Isohydroxamic Acids

Isohydroxamic acid-based HDAC inhibitors have been extensively studied and are widely used due to their ability to inhibit nearly all Zn^2+^-dependent HDACs belonging to Classes I, II and IV. These inhibitors are known to be broad-spectrum inhibitors and are recognized as highly effective in regulating gene expression, cell differentiation and cell death. Unfortunately, this wide-ranging inhibition also gives rise to several adverse side effects. Despite these drawbacks, these drugs show potent antitumor effects and are frequently incorporated into combination therapies with various anticancer agents, amplifying their antitumor efficacy [142]. The hydrophobic channel of HDAC plays a crucial role in its physiological function by accommodating the acetyl-lysine side chain of the substrate. Zn^2+^ forms a five-tooth chelate at the bottom of the channel. However, when the isohydroxamic acid of HDAC inhibitors is present, the hydrophobic channel becomes occupied by the inhibitor’s hydrophobic linker competitively, while the ZBG of the inhibitor is chelated the zinc ion. Specifically, the isohydroxamic acid group, beyond forming a strong diphthong chelate with Zn^2+^, can be involved in oxygen bonds with His142, His143 and Tyr306. Thus, the isohydroxamic acid group acts as a ZBG and shows several advantages, such as unexacting synthesis, excellent in vitro stability, strong zinc binding and elevated solubility [143]. However, isohydroxamic acid groups show some negative aspects to consider. They act as a non-selective ZBG, also binding to other zinc-dependent enzymes, such as aminopeptidases, matrix metalloproteinases and carbonic anhydrases, with the appearance of unwanted side effects. Furthermore, hydrolysis and glucuronidation can adversely affect isohydroxamic acid by impairing its pharmacokinetic properties and reducing its effectiveness in biological systems [144]. Additionally, the linker domain of isohydroxamic acid can vary, featuring either linear or cyclic structures and saturated or unsaturated configurations. The flexibility of linear linkers enables them to interact more effectively with the surface amino acid residues, and this is crucial for HDAC activity. Due to this, in the development of HDACis, more complex cap structures, such as branching caps, are commonly employed. These cap structural domains usually consist of hydrophobic groups, specifically aromatic moieties. By incorporating these cap structures, the inhibitors can be optimized for their potency and selectivity towards HDAC enzymes [145].

In 2006, the US Food and Drug Administration (FDA) approved the clinical use of SAHA (Suberoylanilide Hydroxamic Acid) for the treatment of the cutaneous T-cell lymphoma. SAHA, also known as Vorinostat, was developed by Merck and represented the first approved HDACi [146]. This compound has shown promise in treating hematologic cancers, including diffuse large B-cell lymphoma, follicular lymphoma, and mantle cell lymphoma. In solid tumors such as prostate and pancreatic cancers, SAHA inhibits the Akt/FOXO3a signaling pathway, promoting apoptosis in prostate cancer cells [147]. SAHA also induces autophagy in tumor cells and helps prevent acute graft-versus-host disease. However, its broad-spectrum inhibition leads to significant side effects, including fatigue, diarrhea, anorexia, bone marrow suppression, and thrombocytopenia [148]. Recent research aims to improve the selectivity of HDAC inhibitors or develop new ones based on SAHA’s core pharmacodynamic structure. For example, a new analog, C2-R-SAHA, was created by replacing the hydrogen atom at the C2 position of SAHA’s hydrophobic long chain with aliphatic or aromatic hydrocarbons. This analog has shown the ability to enhance its selectivity for HDAC6 and 8 [149]. The molecular docking analysis identified that Class I HDACs possess a more constricted hydrophobic channel compared to HDAC6. As a result, introducing aliphatic hydrocarbons into the hydrophobic chain of SAHA can enhance the obstruction within this catalytic channel, thereby reducing the effectiveness of HDAC1, 2, and 3. On the other hand, modifying the structure by incorporating aromatic, cyclic, or neighboring isohydroxamic acid groups can boost the selectivity for HDAC6, as exemplified by tubastatin A [150]. Interestingly, Trichostatin A (TSA) shares its structure with SAHA, but displays significantly stronger HDAC inhibition activity. The enhanced activity of TSA is primarily due to its bridging region, which contains a diene and an R-type methyl group. However, this alone does not fully account for TSA’s potency. The arylamine ring in the surface recognition region likely contributes significantly to its effectiveness by interacting with amino acid residues within the enzyme’s active site [151].

### 2.2. Benzamide Derivatives

Benzamide inhibitors are a new promising class of HDACis, characterized by enhanced selectivity and, thus, by reduced side effects. Specifically, they showed a higher selectivity towards HDAC1 and 2 than conventional isohydroxamic acid counterparts thanks to their unique N-(2-aminophenyl) benzamide pharmacodynamic group. Through a molecular docking study, Bass et al. suggested that benzamide inhibitors exhibit a distinct binding mode to histone deacetylase-like protein (HDLP), differing from isohydroxamic acid analogs [152]. Interestingly, their binding does not involve interactions with Zn^2+^. According to the docking findings, benzamides interact with the two benzene rings of Phe141 and Phe198 residues, narrowing the active pocket and obstructing the channel of the N-terminal Lys acetylation side chain of histone, the physiological substrate of HDAC. Such orientation allows for the formation of a hydrogen bond with either Tyr91 or Glu92, while the intermediate benzene ring adopts a sandwich structure with Phe141 and Phe198. This specific binding confers consistent selectivity to benzamide-based inhibitors compared to isohydroxamic acid inhibitors targeting Zn^2+^, thereby reducing toxicity.

The 3D structures of HDAC2 inhibitor complexes disclose the arrangement of the HDAC2 active site, comprising an approximately 8Å-long hydrophobic channel with the catalytic site containing the Zn^2+^ and an adjacent inner cavity termed the “foot pocket,” spanning approximately 14 Å. During inhibition, benzamide inhibitors deeply penetrate this cavity, with the o-amino group and carbonyl oxygen participating in Zn^2+^ chelation. Conversely, one portion of the aromatic ring fits into the catalytic “foot pocket,” leading to a rearrangement of residues to accommodate the aryl group. In contrast, the structural characteristics of SAHA hinder its access to the catalytic foot pocket, explaining its lack of specificity in inhibiting HDAC2 [153]. Intramolecular hydrogen bonding can influence the effectiveness of benzamide inhibitors over time, unlike SAHA, which has a Zn^2+^ chelating group at the top of its molecule. This eliminates the need for extensive protein rearrangement or the breaking of internal ligand hydrogen bonds during the formation of drug-target complexes. The isohydroxamic acid of SAHA can directly bind to Zn^2+^ at the bottom of the hydrophobic channel and replace the bound water, resulting in rapid binding kinetics for ligands containing isohydroxamic acid esters. Benzamide inhibitors, however, must break their intramolecular hydrogen bonds before chelating with Zn^2+^. Furthermore, their large molecular size and curved hydrophobic channels limit their ability to rapidly bind the zinc ion [47].

Chidamide represents the pioneering oral inhibitor of histone deacetylase with subtype selectivity. It has been approved for clinical trials by the State Food and Drug Administration (China Food and Drug Administration, CFDA) [154]. Chidamide falls under the category of benzamide histone deacetylase subtype-selective inhibitors and it is characterized by its distinctive chemical structure. Known chemically as N-(2-amino-4-fluorophenyl)-4-[1]benzamide, Chidamide exhibits potent antitumor efficacy associated with low cytotoxicity relative to its counterparts. Its primary targets include subtypes 1, 2 and 3 of Class I HDACs and subtype 10 of Class IIb [155]. Moreover, Chidamide can induce the differentiation of tumor stem cells and reverse epithelial-mesenchymal phenotypic transformation (EMT) in tumor cells, thereby reinstating drug sensitivity in resistant tumor cells and impeding tumor metastasis and recurrence. This mechanism is attributed to its inhibition of relevant HDAC isoforms, elevation of chromatin histone acetylation levels, and initiation of chromatin remodeling, consequently inducing epigenetic alterations that disrupt the tumor cell cycle and promote apoptosis. Also, Chidamide demonstrates modulatory effects on cellular immunity, enhancing the activity of natural killer (NK) cells and antigen-specific cytotoxic T cells (CTLs) in mediating tumor cell elimination.

### 2.3. Cyclic Peptides 

Cyclic peptides, the most structurally intricate class of HDAC inhibitors, can be categorized into two groups based on the presence of the 2-amino-8-oxo-9, 10-epoxy-decanoyl (Aoe) moiety. In the first group, including cyclic peptides with the Aoe moiety, trapoxin A, trapoxin B and WF-3161 can be mentioned. Apicidin and depsipeptide are examples of the second group, consisting of cyclic peptides without the Aoe moiety. Both peptide groups bind to HDACs similarly to isohydroxamic acids but with distinct mechanisms [156]. The spatial arrangement of Aoe-containing cyclic tetrapeptide macrocycles exhibits a configuration with D-amino acids and cycloamino acids, a spacer region adjacent to the amino acids, and numerous internal hydrogen bonds, resulting in a constricted 12-membered cyclic structure. Indeed, the presence of D-configured amino acids appears crucial for tight binding to the cap activation site edge. Some Aoe-containing inhibitors, for HDAC binding, require an epoxy keto group, a large cyclic peptide structure capable of binding to the duct entrance “groove,” a keto carbonyl group for interacting with Zn^2+^ and polar amino acids within the HDAC channel, and an epoxy group to alkylate the HDAC active site, thereby causing irreversible enzyme inhibition. Notably, substituting the epoxy keto group with isohydroxamic acid renders HDAC inhibition reversible [157]. Cyclic peptide HDAC inhibitors predominantly employ larger cyclic peptide structures as Cap groups. FK228, extensively studied, does not adhere to the classical pharmacophore model of HDAC. In vivo, hydrolysis is needed to free the thiol component of the zinc-chelating group, allowing for effective zinc ion binding for enzyme inhibition. The larger cap group improves interaction with peripheral amino acids, thereby enhancing target affinity [158]. Also, these inhibitors exhibit subtype selectivity for the HDAC family, showing potent inhibitory activity for Class I HDACs while poorly inhibiting Class IIb HDACs, particularly HDAC6. Thus, this selectivity offers insights into designing selective HDAC inhibitors. However, the design and synthesis of these inhibitors face challenges due to the complexity and poor drugability resulting from their large molecular skeleton and mass. 

In 2012, the US FDA approved romidepsin (FK228), an atypical HDACi targeting Class I, for treating cutaneous T-cell lymphomas (CTCL) and peripheral T-cell lymphomas [159]. It is derived from Gram-negative pigmented bacillus No. 968 and features a caged bicyclic phenolic peptide structure with uncommon disulfide bonds, which become activated in human cells following metabolism [160]. FK228, a precursor drug, exhibits greater stability than its reduced form, Red-FK228. The disulfide bond facilitates efficient diffusion across the cell membrane. This process liberates the Red-FK228 free sulfhydryl group, which then interacts with the Zn^2+^ active site, inhibiting HDAC from binding to its substrate.

### 2.4. Future Perspectives and PROTACs

Despite the considerable progress made, numerous unresolved issues in the exploration of HDAC inhibitors (HDACis) remain. Primarily, the majority of HDACis currently available are broad-spectrum inhibitors. In fact, these compounds compete for Zn^2+^ within the enzyme active site, lacking specificity towards distinct isoforms. It is possible to achieve a degree of selectivity for HDAC isoforms by interrupting specific HDAC activities essential for protein–protein interactions [161]. As previously highlighted, HDAC1, 2, and 3 are subunits within multiprotein complexes localized in the nucleus, and dislodging HDACs from these complexes significantly decreases enzyme activity. Thus, inhibiting the assembly of these complexes can partially block HDAC activity. Inositol phosphate is a well-conserved regulatory factor found within a complex of multiple proteins. It has a significant impact on enzyme activity. Indeed, by interacting with arginine residues close to the active site access, inositol phosphate is crucial for forming the complex and activating the enzyme. Therefore, the disruption of this interaction may intensify the inhibitory effect on HDAC1, 2 and 3, which can have negative consequences on enzyme activity.

**Figure 8:**
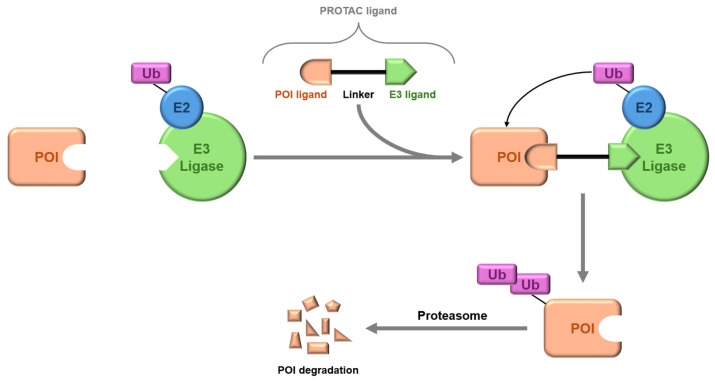
Schematic diagram of a PROTAC, as an innovative approach to improve selectivity for a specific HDAC isoform.

HDAC1, 2, and 3 are components of multiprotein complexes found in the nucleus, and their displacement greatly reduces enzyme activity. Inositol phosphate, a key regulatory factor in these complexes, is essential for their formation and enzyme activation by interacting with arginine residues near the active site. Interrupting this interaction can enhance the inhibition of HDAC activity [162]. HDAC inhibitors commonly use groups such as isohydroxamic acid or carboxylic acid to bind Zn^2+^ in HDACs, but these groups can also target other metalloenzymes, leading to toxicity and limiting their clinical use [163]. To improve therapeutic outcomes and minimize side effects, combination therapies with HDAC inhibitors are being explored. HDAC-PROTACs represent a promising approach by selectively degrading HDACs (Figure 8), reducing off-target effects, and potentially overcoming drug resistance [164,165,166,167,168,169,170,171,172]. PROTACs have been shown to selectively degrade specific HDACs and other proteins, which could enhance the effectiveness of cancer immunotherapies. However, creating effective HDAC-PROTACs is complex due to factors like linker length, E3 ligase selection, and cell type specificity. A more detailed discussion on the development of HDAC-PROTACs is available in the review by Patel et al. [173]. Despite these challenges, HDAC-PROTACs hold significant promise for clinical applications, with ongoing research aimed at enhancing their selectivity and therapeutic potential in cancer and other diseases [174,175].

In addition to isoform selectivity, HDAC1, HDAC2, and HDAC3 are present in vivo within seven distinct corepressor complexes, introducing an additional layer of complexity [166]. These different corepressor complexes play distinct physiological roles in cells, suggesting that targeting specific HDAC-containing corepressor complexes may be crucial for discovering new HDAC therapies with better efficacy and fewer side effects [167]. The future contributions of Proteolysis Targeting Chimeras (PROTACs) in this area are of particular interest. PROTACs, designed to degrade target proteins, generally include three components: a ligand to bind the protein of interest (POI), a ligand to trigger protein degradation (commonly an E3 ligand), and a linker covalently connecting these two ligands (Figure 8). Through the polyubiquitination of lysine amino acids on the POI, PROTACs facilitate eventual degradation by the proteasome. This ubiquitin transfer to the POI relies on the protein–protein interaction between the POI and E3 ligase mediated by the PROTAC [168].

For instance, the pan-BET inhibitor JQ1 functions broadly across BET proteins; however, when JQ1 is included in a PROTAC, it facilitates the specific degradation of BRD4 while sparing BRD2 and BRD3 [169]. Foretinib, a pan-kinase inhibitor, exhibits reduced kinase binding when functionalized into a PROTAC, indicating altered specificity [170]. Additionally, PROTACs’ ability to modify binding affinities and selectivity highlights their dynamic role in protein degradation, as evidenced by studies on p38 isoforms [171]. 

The remarkable ability of PROTAC-mediated degradation to alter protein selectivity has attracted researchers seeking to modulate HDAC activity. Notably, the first PROTAC targeting a histone deacetylase enzyme, NAD^+^-dependent SIRT2 [172], and the first zinc-dependent HDAC-targeting PROTAC for HDAC6 signify significant advancements in the field [174]. While approximately 20 PROTACs are progressing through or are already in clinical trials, none currently target HDACs; nevertheless, several studies have highlighted the great potential of this challenging approach. For instance, in a proteomics investigation by Xiong et al., HDAC degradation was explored with 48 PROTACs varying in HDAC ligand, linker length, and E3 ligand, revealing HDAC1, HDAC2, and HDAC9 as the least frequently degraded zinc-dependent HDAC isoforms, while HDAC6, HDAC8, and HDAC3 were most frequently degraded [175]. Thus, generating selective PROTACs for HDAC1 and HDAC2 posed challenges, given the susceptibility of the other Class I isoform (HDAC3) and HDAC6 to PROTAC-mediated degradation. Nevertheless, some PROTACs demonstrated selectivity in degrading specific HDAC isoforms, like HDAC3, exemplified by the synthesis reported by Xiao et al. of an HDAC3-selective degrader exhibiting significant potency in breast cancer cells [175,176,177,178]. Moreover, several studies have investigated the PROTAC-mediated degradation of HDAC8 [179,180,181,182,183], highlighting its propensity for degradation alongside HDAC3 and HDAC6, with Chotitumnavee et al. developing a selective HDAC8 PROTAC that outperformed its parent inhibitor in compromising cell viability [180].

Within the Class IIa enzymes, only HDAC4-selective PROTACs have been documented thus far, with Macabuag et al. pioneering the development of these PROTACs to explore the role of HDAC4 in Huntington’s Disease [184]. Their study introduced two sets of isoform-selective PROTACs, one incorporating a hydroxamic acid-based inhibitor linked to a VHL E3 ligase ligand via three different PEG lengths, and the other based on a trifluoromethyl oxadiazole HDAC inhibitor [185,186]. Both sets demonstrated dose-dependent degradation of HDAC4 in Jurkat E6-1 cells while sparing HDAC1, HDAC5, HDAC7, and HDAC9. 

Meanwhile, among the eleven zinc-dependent HDAC enzymes, PROTACs targeting HDAC6 for degradation have been extensively reported, suggesting its particular susceptibility to proteasome-mediated degradation by PROTACs. This susceptibility might be attributed to the zinc finger ubiquitin-binding domain that characterizes this isoform [187].

Following K. Yang et al., who discovered selective HDAC6 degradation using a PROTAC incorporating a pan-HDAC inhibitor as the HDAC ligand [174], An et al. drew inspiration from the selective HDAC6 inhibitor Nexturastat A to design PROTACs targeting HDAC6 [188]. The PROTAC, incorporating Nexturastat A as the HDAC ligand and pomalidomide as the E3 ligand, demonstrated impressive efficacy in the B lymphoblast MM.1S cell line, with no degradation of HDAC1, HDAC2, or HDAC4. Additionally, Cao et al. devised a structurally unique HDAC6-targeting PROTAC based on the natural product indirubin as the ligand to engage HDAC6, revealing that shorter PEG linkers containing one PEG unit were notably more effective at HDAC6 degradation than longer PEG linkers [189].

Concerning PROTACs targeting Class III HDACs, J.Y. Hong et al. introduced a new SIRT2 inhibitor, incorporating it into two PROTACs [190], both effectively degrading SIRT2 within a concentration range of 0.5–10 mM in MCF7 and BT-549 cell lines. One of these PROTACs demonstrated the capability to reduce SIRT2 deacetylase and defatty-acylation activity in cells, whereas the SIRT2 inhibitor from which the PROTAC derives was not capable of reducing SIRT2 defatty-acylation activity, showcasing an advantage over the sole inhibition of SIRTs’ catalytic active site.

HDAC11, the sole HDAC isoenzyme in Class IV discovered in 2002, possesses significant fatty-acid deacylase activity and is recognized as a potential target for metabolic disorders [191,192,193]. Despite the existence of selective inhibitors for HDAC11 [194], no PROTACs targeting its degradation have been reported to date.

PROTACs exhibit the potent and selective degradation of individual HDAC isoforms, serving as valuable chemical probes for studying HDAC biology. By targeting HDACs for degradation via the proteasome, researchers can explore their biological roles beyond enzymatic function alone, potentially leading to novel therapeutic applications. While some HDAC-targeting PROTACs show enhanced efficacy in compromising cancer cell viability compared to HDAC inhibitors, others exhibit reverse effects, particularly against pan-HDAC inhibitors. However, future investigations should explore the therapeutic potential of HDAC-targeting PROTACs beyond cytotoxicity, considering their ability to enhance antitumor immune responses and overcome drug resistance in cancer immunotherapies, as evidenced by recent studies [195,196]. Ongoing clinical trials investigating combination therapies of HDAC inhibitors with immune checkpoint inhibitors suggest a promising avenue for the therapeutic application of PROTACs targeting HDACs [196].

## 3. Animal Research and Clinical Trials with HDAC Inhibitors

Animal studies investigating HDAC inhibitors have provided valuable insights into their potential therapeutic applications across various diseases [197]. These studies often involve the administration of HDACis to animal models, such as mice or rats, to evaluate their efficacy, safety and mechanism of action [18,198]. By examining the effects of HDACis on disease progression, biomarkers and physiological parameters in these models, researchers can better understand their pharmacological properties and potential clinical benefits. Furthermore, animal studies allow for the exploration of optimal dosing regimens, routes of administration and combination therapies, which are essential for translating preclinical findings into successful clinical trials. Overall, animal studies play a crucial role in advancing our knowledge of HDACis and their therapeutic potential in treating a wide range of diseases [199,200,201,202]. On the other hand, clinical studies investigating HDACis in humans aim to assess their safety, efficacy and tolerability across various diseases, providing essential data for their potential use as therapeutic agents. The clinical trial information of some drugs is summarized in Table 5. The majority of pan-HDAC inhibitors are currently undergoing Phase II/III clinical trials, with several demonstrating promising outcomes [203,204,205]. A Phase II clinical study investigating Panobinostat revealed a substantial therapeutic benefit in treating multiple myeloma. This effect might be further enhanced by combining it with other related medications [203]. The next generation of selective inhibitors is expected to achieve heightened effectiveness in treating hematologic and solid tumors, broadening the range of diseases addressed by HDACis. With enhanced targeting mechanisms, these inhibitors will also help reduce side effects, bringing us closer to a future where cancer treatment is more effective and less harmful [206]. Beyond cancer, HDAC inhibitor drugs have demonstrated effectiveness in various other conditions. Vorinostat, for instance, is presently undergoing clinical trials for Alzheimer’s disease (NCT03056495). In neurodegenerative disorders, it is anticipated to be utilized for frontotemporal dementia resulting from progranulin deficiency, albeit with a need for further efficacy enhancement [207]. Significantly, a growing number of drugs have exhibited therapeutic potential in addressing HIV infection. This outcome could be linked to the cellular autophagy and immunomodulatory functions mediated by HDACs. With the expansive range of genetic regulatory functions attributed to HDACs, an escalating number of clinical trials for various diseases have progressed to the Phase II stage, offering further avenues for research in both clinical and preclinical settings. However, it is concerning that numerous adverse events persist in the clinical trials of marketed HDACis in different cancer types [208,209]. Despite the improved metabolic half-life and assured oral bioavailability of the latest clinical candidate, Pracinostat (Figure 4), the completion of a co-administration trial (NCT03848754) has revealed prevalent toxicities including nausea/vomiting (63%), anorexia (50%), hypokalemia (50%) and rhinorrhea accompanied by neutropenic symptoms [210]. Although present-day HDACis demonstrate potential in inhibiting cell proliferation in vitro, their lack of selectivity results in undesirable side effects, including off-targeting. This can lead to the harmful attack of healthy cells, causing significant toxic reactions. Furthermore, the increase in HDAC drug trials has raised worries about drug resistance [211,212,213]. This has led to the understanding that resistance to HDAC inhibitors involves many factors.

## 4. Computational Studies on HDACs

### 4.1. Molecular Modeling

**Figure 9:**
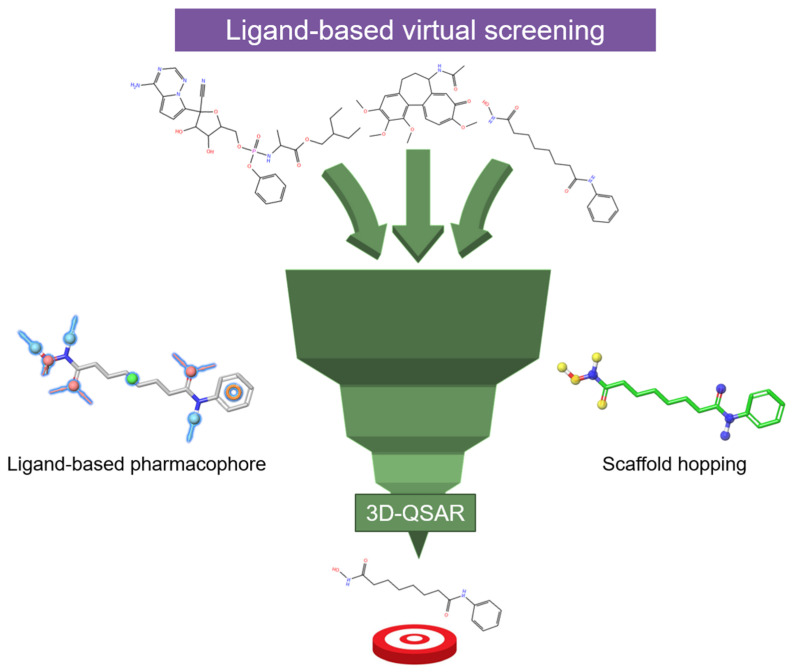
Schematic representation of some ligand-based (LB) approaches for the virtual screening of large databases to discover new HDACis. The features of the ligand-based pharmacophore based on Vorinostat are illustrated as follows: the aromatic ring is represented by an orange ring, the hydrophobic feature is depicted as green spheres, and H-bond acceptors and donors are shown as red and light-blue spheres associated with arrows, respectively. In the scaffold hopping analysis, blue and yellow spheres denote the H-bond donor/acceptor moiety of Vorinostat. Specifically, blue spheres are designated as optional matches.

**Figure 10:**
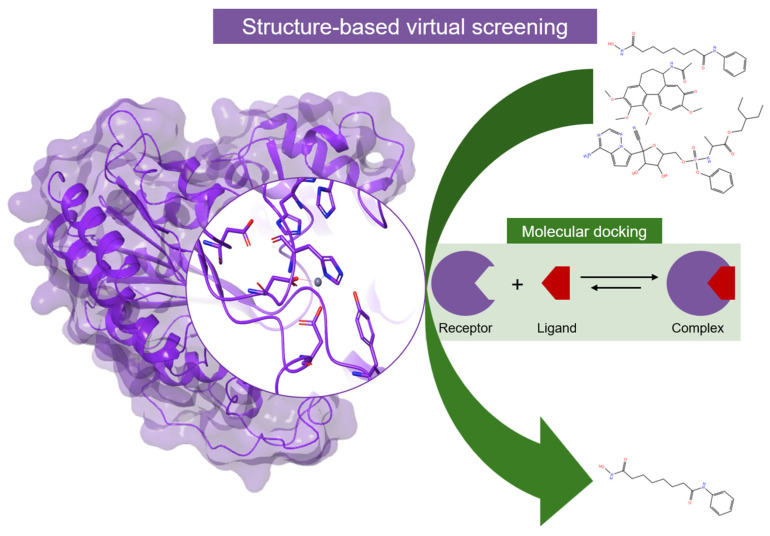
Schematic representation of some structure-based (SB) approaches for the virtual screening of large databases to discover new HDACis. HDAC8 is represented as violet surface and ribbons, while the main residues of the catalytic site are shown as violet thin tube.

Over the last two decades, several computational approaches have been employed to find HDAC inhibitors with enhanced potency and/or selectivity. The main purpose is to simplify the search process, reducing the search space and ensuring the identification of the most promising compounds with desired activities. Computational techniques, including ligand-based approaches (Figure 9) such as scaffold hopping, 3D-QSAR, and pharmacophore modeling, as well as structure-based methods like structure-based virtual screening/molecular docking (Figure 10) and fragment-based ligand design, have proven instrumental in developing HDAC inhibitors with targeted activity. 

**Figure 11:**
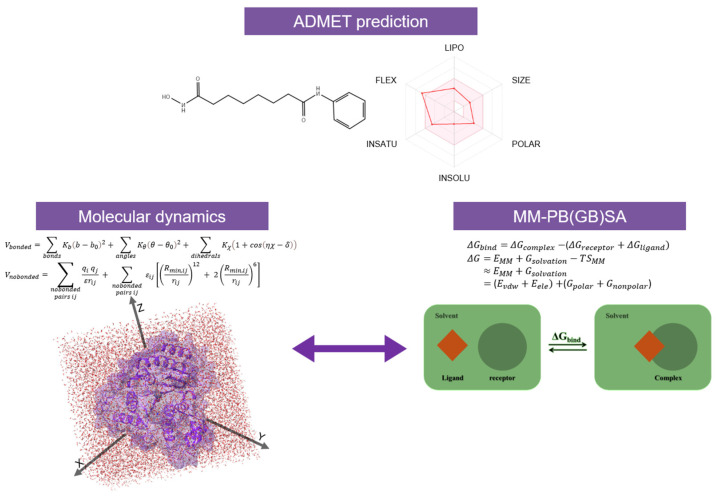
Schematic representation of ADME prediction, Molecular Dynamics and MM-PBSA/MM-GBSA binding energy calculations. HDAC8 structure is depicted as violet surface and ribbons situated within a water box.

Thus, the potential *hits* identified undergo further validation through structure-based assessments, utilizing techniques such as molecular dynamics (MD) simulations coupled with MM-PBSA/MM-GBSA binding energy calculations (Figure 11). The MM/PBSA and MM/GBSA methodologies estimate the free energy of ligand binding to biological macromolecules, serving as intermediary tools bridging empirical scoring (e.g., docking and scoring) and rigorous alchemical perturbation (AP) methods [214,215]. The application of combined computational approaches in HDACi rational design allows us to significantly limit the risk of false positive *hits*. Moreover, such methodologies increase the possibility of retrieving specific inhibitors by employing different filters and scoring functions.

Scaffold hopping strategies, along with molecular docking, have been employed in various studies for the design of HDAC inhibitors. Usually, the majority of newly developed compounds with improved potency and/or desired selectivity are achieved through modifications in the three distinct structural regions of HDAC inhibitors. An exemplary demonstration of this strategy is seen in a series of quercetin-containing hydroxamic acid derivatives. These derivatives were synthesized by altering quercetin in both the cap and the linker regions, while their ability to bind HDAC was initially assessed in silico [216]. Given the recognized pan-HDAC inhibition activity of resveratrol, a combination of scaffold hopping, molecular docking and ADME prediction was utilized to create a set of resveratrol analogs. These prospective inhibitors were subsequently subjected to additional validation via MD simulations and in vitro enzyme inhibition assays targeting HDAC1 and HDAC2 [217,218]. The scaffold hopping strategy was also useful for the discovery of some hybrids bearing 1H-indazol-3-amine and benzohydroxamic acids with dual HDAC/EGFR1 inhibitory activity against breast cancer line MCF-7 [219], this approach also led to the identification of a new class of Aminotetralin-based inhibitors selective for HDAC6 and HDAC8, demonstrating significant inhibitory effects on neuroblastoma BE(2)C cells [220]. Notably, two HDAC6-selective inhibitors were developed for the first time, incorporating 2-mercaptoquinazolinone as the cap moiety. This design strategy involved altering the surface recognition group by using the quinazolinone core as the cap and fine-tuning the linker, while maintaining hydroxamic acid side chains at either the C-2 or N-3 position [221]. In a recent investigation, HDAC inhibitors featuring 4-acyl pyrrole caps were employed as a scaffold for developing potent hybrid inhibitors that target both bromodomain and extra-terminal (BET) proteins as well as HDACs [222]. Considering all these findings, the combined use of scaffold hopping with molecular docking emerges as a critical computational strategy for formulating HDAC inhibitors with the desired pharmacological characteristics.

As previously stated, HDAC inhibitors traditionally feature a pharmacophore composed of three fundamental elements: a cap, a linker region, and a ZBG [223]. Modifications in the ZBG aim for increased potency, while variations in the cap and linker regions are designed to enhance selectivity for particular HDAC isoforms. Pharmacophore models can be classified as structure-based, which are derived from the complexes of proteins and ligands, or ligand-based, which are based on the structures of existing HDACis. These models undergo validation to ensure their efficacy in distinguishing active and inactive compounds, often through methods like Receiver Operating Characteristic (ROC) analysis or inactive compounds (decoy) testing [224]. For instance, in the search of potential selective inhibitors targeting HDAC2, a total of 300,000 compounds sourced from the Asinex, National Cancer Institute (NCI), and Maybridge databases were screened using e-pharmacophore modeling [225]. Also, the interaction between benzamide MS-275 and HDACs was investigated by means of a 3D chemical feature-based QSAR pharmacophore model [223]. A potent HDAC3 inhibitor was identified by Kumbhar et al., by using an integrated computational screening strategy, which included ligand-based pharmacophore modeling, MD simulation, and MM-PBSA calculation methods [226]. It is noteworthy that combining MD simulations with energetically optimized structure-based pharmacophores (e-Pharmacophores) was useful in the rational design of potential HDAC inhibitors, as validated by MM-GBSA binding energy calculations [227]. Thanks to dynamic pharmacophore models, it became possible to address the issue concerning the flexibility within the protein’s active site. Specifically, this method was utilized to seek out potential inhibitors of HDAC8 by generating structure-based pharmacophore models from various conformations obtained through MD simulations [228]. 

Quinoline has been used as a cap group in the development of many HDAC inhibitors [229,230,231,232]. Among them, the quinoline-based HDAC inhibitor CHR3996 has completed a Phase I clinical study [233,234]. In 2017, Chen et al. designed several quinoline-based HDAC inhibitors whose binding mode was found to be the same as that of traditional HDACis. Interestingly, the authors observed that the eight positions of quinoline did not occupy the pocket, thus encouraging them to modify such positions in order to improve the activity and selectivity [235]. Therefore, in a more recent study, they designed and synthesized a new series of 8-substituted quinoline-2-carboxamide derivatives and identified a very potent compound (IC_50_ = 0.050 µM) that exhibited 3-fold greater HDAC inhibitory activity compared to the known HDAC inhibitor Vorinostat, with low toxicity against normal cells [236]. 

More recently, Gao and co-workers developed and synthesized novel HDAC inhibitors derived from the β-elemene scaffold [237]. β-elemene is specifically a sesquiterpene used in the treatment of lung cancer, pancreatic cancer, gastric cancer, breast cancer, bladder cancer, and malignant brain glioma [238,239,240,241,242,243]. Most of the prepared compounds, whose binding mode was fully investigated by means of molecular docking analyses, showed potent inhibitor activities against HDACs and significant inhibitory effects on the proliferation of K562 and MV4-11. Two derivatives demonstrated excellent in vitro antienzyme (IC_50_ values of 22 nM and 9 nM for HDAC1 and 8 nM and 14 nM for HDAC6, respectively) and broad spectrum in vitro antiproliferative activities (IC_50_ values ranging from 0.79 to 4.42 mM against K562, MV4-11, HEL, SU-DHL-2 and WSU-DLCL-2 cell lines) and, among them, one was found to induce cell apoptosis and to exhibit antitumor activity in the WSU-DLCL-2 xenograft mouse model, without significant toxicity.

Initial investigations into HDAC inhibitors utilized comparative molecular field analysis (CoMFA) and comparative molecular similarity indices analysis (CoMSIA) for the design of novel HDAC inhibitors [244]. Afterwards, various QSAR analyses, including 3D-QSAR and multi-QSAR modeling, facilitated the discovery of potent HDAC inhibitors. As for indole amide analogs, a 3D-QSAR analysis was conducted on HDAC1 to identify the compounds with the highest predicted inhibitory activity [245,246]. Moreover, QSAR classification models, such as k-nearest neighbors (kNN) and neighborhood classifier (NEC), were employed to predict potential HDAC8 inhibitors [247]. Recent studies utilized 3D-QSAR analysis to design potential selective inhibitors of HDAC6 and explore selective HDAC8 inhibitors through QAAR studies [248,249]. A multi-QSAR modeling study successfully identified potent HDAC8 inhibitors [246]. Recent approaches involved 3D-QSAR analysis to design potential selective HDAC6 inhibitors [249]. QAAR studies, utilizing DFT-based calculation and molecular dynamic simulation, explored selective HDAC8 inhibitors [248]. Similarly, QAAR and molecular docking led to the discovery of selective HDAC8 inhibitors with antiproliferative activities [250]. Also, as for isoform 1, the 3D-QSAR model was utilized to predict potential HDAC1 inhibitors with high activity. Additionally, protein–ligand interactions were refined using induced fit docking (IFD), and molecular dynamics (MDs) simulations combined with MM-GBSA calculations were also utilized to enhance the accuracy of the analysis [251]. To overcome the limitations of 3D-QSAR, researchers advanced to 4D-QSAR models that integrate molecular state ensemble averaging [251].

Thanks to the abundance of crystal structures in HDACs, designing inhibitors based on their structure has become more feasible. Currently, the crystal structures of various human HDAC classes are available on the Protein Data Bank (Figure 2, Figure 4 and Figure 5) (https://www.rcsb.org/), as previously described [252]. For other isoforms, homology models have been built to study their potential inhibitors. Notably Hsu et al. utilized homology modeling to construct human HDAC5 and 9 models. Utilizing these models and the crystal structures of HDAC4 and HDAC7, researchers conducted a virtual screening of the NCI compound library based on structure and molecular docking. This approach aimed to identify potential inhibitors of Class IIa HDACs. The identified compounds were then tested against HeLa nuclear Class II HDACs to find those with selective inhibition for Class IIa HDACs [253]. In 2019, Ibrahim Uba et al. applied a homology modeling study for human HDAC10, exploiting the crystal structure of HDAC10 derived from zebrafish (PDB code: 5TD7). Then, this theoretical model was submitted to structure-based virtual screening, MD simulations and ADMET prediction techniques, for identifying potential HDAC10 inhibitors [254]. In the study by Géraldy et al., an additional homology model of human HDAC10 was introduced, emphasizing the significance of a crucial hydrogen bond formation between a nitrogen atom in the cap group and the gatekeeper residue Glu272 in influencing HDAC10 binding [254]. Therefore, the accessibility of crystal structures for human HDACs has greatly improved the effectiveness of structure-based inhibitor design. Moreover, when biological activity data are unavailable, researchers have been using a method known as MM-GBSA/MM-PBSA ligand binding affinity calculations to determine the effectiveness of identified *hits*. For example, Sixto-López et al. used molecular docking, MD simulations and MM-GBSA energy calculations to design hydroxamic acid derivatives with potent inhibitory activity against HDAC1, HDAC6 and HDAC8. YSL-109 emerged as the most active compound against hepatocellular carcinoma, neuroblastoma and breast cancer [255]. Also, novel methods including e-pharmacophore modeling, structure-based virtual screening and MD simulations combined with MM-GBSA ligand binding energy calculations have been utilized in the quest to identify potent inhibitors of HDAC2 [225]. A thorough analysis was performed to discover potential inhibitors of Class IIa HDACs using a virtual screening process. This involved the usage of MD simulations and MM-PBSA to determine the ligand-free energy of binding. The compounds that exhibited optimal results based on these calculations were ultimately chosen as the final *hits* [256]. Similarly, a “multi-layer virtual screening workflow” was developed for identifying inhibitors selective for the HDAC6, demonstrating significant anticancer activity [257]. In a recent development, the screening of an in-house compound library using a combined approach of structure-based and pharmacophore modeling led to the identification of a compound with nanomolar activity against HDAC1, HDAC3, and HDAC6. This compound displayed superior inhibitory effects compared to Vorinostat and exhibited potential in impeding the growth of solid cancers [258].

In summary, the computational methodologies mentioned above complement each other, providing multiple layers of filtration essential for successful hit discovery. Typically, pharmacophore modeling and/or 3D-QSAR modeling serve as the foundational models utilized in pharmacophore-based/ligand-based virtual screening. Following the identification of potential hits, structure-based virtual screening and molecular docking are utilized to anticipate binding poses and affinities. The most promising candidates are evaluated through MD simulations to assess the stability of their ligand binding modes, MM-GBSA/MM-PBSA calculations are commonly integrated with MD simulations.

More recently, various studies have been focused on the elucidation of the simultaneous inhibition of two or more targets involved in critical pathways related to cancer progression. For example, Duan and co-workers applied computational and SAR approaches, and identified a series of pyridazinone-based PARP7/HDACs dual inhibitors whose in vitro and in vivo activities were evaluated. In particular, a hydroxyl propenamide derivative was reported as a potent and balanced dual inhibitor, with an excellent antitumor capability towards lung, B-myelomonocytic leukemia and histiocytic lymphoma cell lines, thus suggesting a relationship between anticancer immunity and HDAC inhibition [259].

Inspired by the synergistic effects of tubulin and HDAC inhibitors in dual targeting cancer therapy and the interaction between proteins, some *o*-aminobenzamide-based dual HDAC/tubulin inhibitors have been reported, which could target tumor tissues more accurately, thus enhancing their efficacy and improving the antitumor effects [260]. In 2022, Yao’s team reported a series of 2ME2 derivatives as dual HDAC/tubulin inhibitors by combining the pharmacophore of a HDAC inhibitor with the 2-methoxyestradiol (2ME2) skeleton [261]. Among them, a compound showed potent dual inhibitory activities on tubulin polymerization and HDAC (IC_50_ values were 0.06 and 0.12 μM of HDAC2 and HDAC6, respectively), as well as exhibited potent antiproliferative activities against MCF-7, MGC-803, HeLa, A549, HepG2 and U937 with IC_50_ values of 0.37–4.84 μM. In addition, the compound also exhibited potent in vitro and in vivo antitumor and antiangiogenic response. Its well-defined binding modes in tubulin (PDB code: 5LYJ) and HDAC2 (PDB code: 4LXZ) helped to explain in detail the high inhibitory potency on tubulin and HDAC2. More recently, novel dual tubulin/HDAC inhibitors were designed and synthesized based on the structure of natural product millepachine, which has been identified as a tubulin polymerization inhibitor [262]. A biological evaluation revealed that a derivative exhibited an impressive potency against PC-3 cells with the IC_50_ value of 16 nM, and effectively inhibited both microtubule polymerization and HDAC activity. Furthermore, the compound induced PC-3 cells apoptosis with a decrease in mitochondrial membrane potential and an elevation in reactive oxygen species levels in PC-3 cells. Additionally, it showed inhibitory effects on tumor cell migration and angiogenesis with favorable drug metabolism characteristics in vivo. Molecular docking analysis provided additional evidence supporting the binding of the identified dual inhibitor to tubulin and HDAC [263]. 

Also, the simultaneous inhibition of Class I phosphoinositide 3-kinases (PI3K) and HDAC has shown promise for treating various cancers [264]. Several PI3K/HDAC dual inhibitors have been disclosed, showing promising anticancer properties [265,266,267,268]. The first PI3K/HDAC dual inhibitor entering into the clinical trials, CUDC-907, was granted the fast-track designation by the FDA for treating relapsed or refractory diffuse large B-cell lymphoma [269]. Recently, Zhang et al. reported a novel series of 4-methylquinazoline based PI3K/HDAC dual inhibitors characterized by a hydroxamic acid moiety as a HDAC pharmacophore [270]. Despite favourable antiproliferative activities against a broad panel of cancer cell lines, these compounds only showed limited in vivo activities largely due to their poor pharmacokinetic properties. Therefore, the same research team incorporated the benzamide moiety as the zinc binding group and, by means of molecular docking and QSAR studies, obtained two potent PI3K/HDAC dual inhibitors for the treatment of acute myeloid leukemia with improved pharmacokinetic properties [271].

### 4.2. Machine Learning

Recently, machine learning has garnered significant attention in the initial phases of drug discovery studies [272]. Machine learning has opened up avenues to explore the vast chemical space beyond the limitations of conventional experimental techniques [273,274]. In medicinal chemistry, a range of machine learning models utilize algorithms including decision trees (DT), random forests (RF), support vector classifiers (SVC), k-nearest neighbors (kNN), Gaussian naive Bayes (GNB), and deep neural networks [274,275,276]. AlphaFold represents a promising machine learning approach, capable of accurately predicting the 3D structure of proteins, even in the absence of closely related structures. However, its models are generated without considering the presence of small molecules, ions, or cofactors, which complicates their direct application in drug design. In a recent study, Baselious and colleagues demonstrated the utility of an optimized AlphaFold model for virtual screening, specifically addressing HDAC subtype selectivity [277]. In the developed multistep screening process, various methodologies were employed, including structure-based pharmacophore screening to filter large databases, ligand docking, pose filtering, and prioritization. This stepwise virtual screening approach successfully identified a hit compound, subsequently validated using an in vitro enzymatic assay. The compound exhibited an IC_50_ value of 3.5 µM for HDAC11 and demonstrated the selective inhibition of HDAC11 over other HDAC subtypes at a concentration of 10 µM. Molecular dynamics simulations confirmed the stability of the initial binding mode, as evidenced by ligand RMSD, RMSF, bidentate chelation of the zinc ion, and interaction stability [278]. 

The absence of an experimental 3D model for HDAC10 poses a challenge to structure-based drug design for selective inhibitors. Consequently, various ligand-based modeling techniques offer a primary avenue to accelerate inhibitor design. In a recent investigation, Bhattacharya and collaborators employed diverse ligand-based modeling approaches on a wide array of HDAC10 inhibitors, leveraging machine learning models to screen for potential HDAC10 inhibitors within a vast chemical database. Furthermore, Bayesian classification and Recursive partitioning models were utilized to identify structural fingerprints governing HDAC10 inhibitory activity. Complementarily, a molecular docking analysis was conducted to elucidate the binding patterns of these identified structural fingerprints within the active site of HDAC10, providing invaluable insights for medicinal chemists in the design and development of effective HDAC10 inhibitors [279]. 

A recent study delved into HDAC8, a protein implicated in cancer progression. While many reported inhibitors targeting HDAC8 feature a hydroxamic acid group, known for its mutagenic potential, Nurani et al. turned to machine learning for drug screening, aiming to uncover alternative compounds devoid of hydroxamic acid while retaining HDAC8 inhibitory activity [280]. In this investigation, the authors devised a predictive model utilizing the random forest algorithm to screen for HDAC8 inhibitors, selected for its superior accuracy on the training dataset inclusive of data augmented by the synthetic minority oversampling technique (SMOTE). Employing the trained RF-SMOTE model, they successfully identified a selective non-hydroxamic acid derivative as an HDAC8 inhibitor, exhibiting an IC_50_ of 842 nM.

Furthermore, the study addressed HDAC1, another pivotal isoform implicated in numerous tumors. Li et al. compiled a dataset comprising 7318 HDAC1 inhibitors and computed four types of molecular fingerprints (MACCS, RDK, ECFP4, and TT fingerprints) to delineate molecular structural features. By calculating Tanimoto coefficients, they ensured a dataset rich in structural diversity, enabling the establishment of 80 classification models across four types of molecule fingerprints using five machine learning algorithms. Additionally, employing the DT algorithm, the authors dissected the structure–activity relationship of HDAC1 inhibitors, identifying certain substructures, such as N-(2-amino-phenyl)-benzamide, benzimidazole, hydroxamic acid with a middle-chain alkyl, and 4-aryl imidazole with a mid-chain alkyl featuring a chiral α carbon, as exerting a significant impact on high activity [281].

In contrast, HDAC6 has emerged as a potential therapeutic target associated with various diseases, notably cancer and neurological disorders, such as Rett syndrome, Alzheimer’s disease, and Huntington’s disease. In a recent study aimed at developing selective and potent HDAC6 inhibitors, Banerjee et al. investigated the structural determinants of quinazoline-cap-containing HDAC6 inhibitors through a combination of machine learning, conventional QSAR analysis, and MD simulation-based binding mode analysis. This integrated molecular modeling approach highlighted the critical role of the quinazoline moiety and its substitutions, as well as molecular properties such as the number of hydrogen bond donor–acceptor functions and the carbon–chlorine distance, in modulating the binding affinity of these inhibitors to HDAC6, thereby influencing their potency. Additionally, the study revealed that substitutions such as the chloroethyl group and bulky quinazolinyl cap group could impact the interaction of the cap function with amino acid residues near the catalytic site of HDAC6, potentially leading to both stabilization and destabilization of the cap function following the occupation of the hydrophobic catalytic site by the aryl hydroxamate linker–ZBG functions [282].

### 4.3. Limitations of Computational Techniques

Advancements in computing technology and algorithmic sophistication have significantly enhanced the capability of executing computationally demanding biomolecular modeling tasks. Computer-aided drug design methods leverage these advancements to improve the accuracy and efficiency of molecular modeling predictions. Particularly noteworthy are the multilayered computational strategies employed in the quest for potential HDAC inhibitors, which harness the predictive capabilities of various scoring functions for effective filtering. For instance, empirical scoring functions are commonly utilized to predict ligand binding pose and affinity in structure-based drug design initiatives [283]. Yet, formulating active compounds or identifying them solely through computational modeling poses a formidable challenge. In certain cases, specific benchmarking techniques are required to manage extensive ligand datasets. Another consideration is the complete flexibility of the protein during molecular docking, which remains a concern even when induced fit and entropy effects do not significantly influence binding [284,285]. Contemporary trends in integrating MM-GBSA/MM-PBSA calculations of ligand binding affinity in the quest for potential HDAC inhibitors aim to enhance the precision of hit selection [226,286]. This is particularly significant considering that in certain studies employing multilayered approaches, the identified *hits* underwent no experimental validation. Overall, the utilization of combined computational methods has demonstrated efficacy in facilitating the design of both class- and isoform-selective inhibitors.

In LBVS, Support Vector Machines (SVMs) are commonly employed for binary property or activity predictions. For instance, they are utilized to differentiate between drugs and nondrugs [287,288], or between compounds with specific activity and those without [289,290]. They are also applied for predicting synthetic accessibility [291] or aqueous solubility. The scores generated by SVM classification have proven effective in ranking database compounds based on their decreasing likelihood of activity [292]. This ranking is often determined by the signed distance between a candidate compound and the hyperplane. Two studies have proposed specialized ranking functions for virtual screening to enhance SVM ranking accuracy, addressing the tendency of SVMs to prioritize classification performance over ranking optimization [293,294].

Various new kernel functions have been introduced for SVMs, including ligand and target kernels, which capture distinct information for a similarity assessment [295,296]. These kernels utilize different metrics such as graph or descriptor similarity for compounds and sequence, or binding site similarity for target proteins. For instance, graph kernels [297] facilitate the overall similarity computation between labeled graphs, eliminating the need to compute or store a vector representation of compounds. However, they entail high computational costs and require parameter tuning.

Decision tree (DT) models offer simplicity in understanding, interpreting, and validating predictions. However, they are prone to high variance, where even slight changes in the data can lead to different split sequences, complicating interpretation. This instability stems from the hierarchical process, where errors in higher splits cascade downward, affecting subsequent splits. Additionally, DT structure is sensitive to minor variations in the training data; small datasets can significantly impact the learning process, while large datasets may induce overfitting. To address these challenges, it is advisable to maintain a moderate training dataset size and a balanced tree structure with a reasonable number of levels. Heuristic approaches can be used to enhance classification accuracy by adjusting subtrees at lower levels. The performance of DTs also relies on selecting splitting attributes sorted by importance, ensuring that the most crucial attributes guide the splits at each level. To mitigate high variance, pruning techniques are commonly employed, utilizing either model complexity parameters or cross-validation. While individual DTs may not yield high-performance models, ensemble methods, such as bagging [297], boosting [298], and stacking [299], outperform individual learners by leveraging the variability among ensemble members, thereby capitalizing on the variance of DTs. In particular, Random Forest (RF) models have shown promise in enhancing the performance of individual DTs in ligand-based virtual screening (LBVS) and have applications in post-docking scoring functions and predicting protein-ligand binding affinity.

In ligand-based virtual screening (LBVS), Bayesian modeling methods are utilized to forecast the likelihood of a compound’s activity based on its descriptor vector. By leveraging known active (A) and inactive (Z) training compounds, these methods estimate conditional probability distributions P(B/A) and P(B/Z) given the representation B, respectively. Consequently, Bayesian classifiers excel in ranking compound databases according to their probability of activity. However, a significant drawback arises when there are substantial conditional dependencies between variables, rendering the naive Bayesian model unsuitable for such scenarios.

The kNN algorithm, a straightforward technique used to predict the class [300], property [301], or rank [302] of a molecule based on its nearest neighbors in the feature space, heavily relies on the local structure of the data. Hence, it is particularly effective for predicting properties with strong locality, such as protein function [303,304]. Despite its intuitive nature, the kNN method does have its limitations. Firstly, since it depends solely on the nearest k neighbors to predict a new compound, it is vulnerable to noisy data. A single misclassified training data point could lead to an incorrect prediction for a new molecule. Additionally, the inclusion of irrelevant descriptors may result in erroneous predictions. Moreover, the predicted value cannot exceed the maximum or minimum activity levels present in the training set.

In the field of medicinal chemistry, Artificial Neural Networks (ANNs) find applications in compound classification, QSAR studies, primary virtual screening of compounds, identification of potential drug targets, and the localization of structural and functional features of biopolymers [304,305]. Originally inspired by the structure and function of the brain, ANNs have evolved into versatile nonlinear regression models [306], offering flexibility in modeling complex relationships. However, one common challenge with ANN simulations is their ‘black box’ nature, where resulting classification models lack interpretability or explanation in physical or chemical terms. Nevertheless, ANNs excel at capturing and modeling nonlinear relationships, which is a significant advantage in many applications.

### 4.4. Achieving Selectivity for Each HDAC Isoform

The selectivity of HDAC inhibitors has emerged as a critical matter for their application in cancer therapy. First-generation HDAC inhibitors, such as Vorinostat, Belinostat and Panobinostat, target multiple isoforms, leading to cellular toxicities due to their hydroxamic acid functional group, as discussed elsewhere [307]. Seeking safer alternatives, the next generation of HDAC inhibitors predominantly aims for class or isoform selectivity, with various studies highlighting alternative zinc-binding groups (ZBGs) exhibiting high inhibitory activity along with selectivity [307]. For instance, imidazole thione-containing molecules have shown promise against HDAC8 [308]. Also, pyrimido[1,2-c][1,3]benzothiazin-6-imines have demonstrated high selectivity [309]. Further research, based on molecular docking and dynamic simulations, allowed for the identification of tropolone derivatives as selective HDAC2 inhibitors [310], while 3-hydroxypyridin-2-thione (3-HPT) was associated with the selective inhibition of HDAC6 [311]. Compounds with benzoylhydrazide as ZBG have exhibited potent inhibitory activity against Class I HDACs, particularly when possessing a 3-carbon-length β-nitrogen alkyl substituent chain [312]. Additionally, trifluoromethyloxadiazolyl moiety has shown selectivity for Class IIa HDACs [313], and 2-substituted benzamide has been used as a ZBG for selective inhibitors of HDAC3 [219]. Notably, an alternative approach proposed by Maolanon et al. focuses on disrupting protein–protein interactions essential for HDAC activity rather than chelating the active site zinc ion [314]. Employing a top-down combinatorial in silico approach, Ganai et al. explored strategies to selectively inhibit HDAC1 and HDAC2 (sequence identity: 94%), revealing distinct pharmacophore features for each isoform. According to the results of pharmacophore analysis, Dacinostat (LAQ824) showed a higher affinity towards HDAC1 when a positive ionizable group was present in the linker region. On the other hand, the ring in the linker region displayed a stronger interaction with HDAC2 [227]. Previously, Abdizadeh et al. designed potent HDAC1 inhibitors by developing biaryl benzamides using a combination of 3D-QSAR and molecular docking techniques [315]. In another study, Cao et al. explored various modifications to the cap and linker regions to enhance selectivity for HDAC3. Meanwhile, Suzuki et al. identified specific compounds with phenyltriazole cap groups that exhibit greater selectivity for HDAC3 when compared to other Class I members [316,317].

HDAC6, although sharing structural similarities with HDAC10, presents slight distinctions compared to Class I HDACs, particularly in active site dimensions and surface residues. A set of compounds with 2-mercaptoquinazolinone as the cap group was designed to target HDAC6 specifically. This approach focused on optimizing the surface recognition element by incorporating the quinazolinone core as the cap, along with modifying the linker structure. At the same time, it maintained the hydroxamic acid side chains at either the C-2 or N-3 positions to ensure their efficacy [221]. Recently, researchers have explored various avenues to design HDAC6-selective inhibitors. For instance, a 3D-QSAR analysis, leveraging the classical pharmacophore of HDAC inhibitors, was employed to devise potential HDAC6-selective compounds [318]. Pharmacophore models, notably HypoGen-based 3D QSAR, based on the crystal structure of HDAC6 (PDB ID: 5EDU), have been instrumental in identifying potent HDAC6 inhibitors and assessing their selectivity. Further assessment of these inhibitors involved MD simulations and analysis of protein–ligand interaction energy (PLIE) [286]. Lastly, in a separate initiative, Moi et al. utilized a cheminformatics approach to develop HDAC6 inhibitors, This interesting approach led to the unexpected discovery of aminotriazole, which exhibited remarkable subnanomolar potency and a notable selectivity for Class I HDACs, specifically HDAC1 and HDAC8 [319].

Another successful virtual screening approach was applied to HDAC7 by our research group. Specifically, in line with our research, and to figure out novel anticancer strategies, an in-house chemical database of extracted molecules from both edible and non-edible mushrooms was adopted [320]. Using structure-based virtual screening (SBVS), ibotenic acid emerged as a potential HDAC7 inhibitor due to its predicted binding affinity. Subsequent in vivo studies revealed that ibotenic acid effectively reduced cellular viability in MCF breast cancer cells, highlighting its potential as a repurposed natural product for cancer treatment.

What is highly interesting is, while single isoform inhibition has been extensively investigated, recent studies highlight the efficacy of dual/multi-targeting HDAC inhibitors in achieving synergistic and enhanced cancer therapy outcomes [321,322].

Therefore, in order to enhance antineoplastic activity and reduce undesirable effects, the world of research is focusing on the pursuit of dual binders capable of simultaneously inhibiting, for example, specific HDAC isoforms and tubulin, the Vascular Endothelial Growth Factor, Tyrosine kinase receptors, Hepatocyte growth factor receptor, Cyclin-dependent kinases, A2A adenosine receptor, Poly ADP-ribose polymerase and others [323].

## 5. In Vitro Validations Using Cell Lines

It is critical to ensure the accuracy and reliability of computational predictions by subjecting them to laboratory experiments for validation. This process verifies computational results through in vitro tests, which allow researchers to evaluate the robustness of computational methodologies and their applicability to real-world biological systems. In vitro validation also provides empirical evidence to support hypotheses and computational insights, thereby improving the credibility and reproducibility of computational studies. By identifying any discrepancies or limitations in computational models, experimental validation drives refinements and improvements for future analyses. Integrating in vitro validation with computational studies is critical in advancing our understanding of complex biological phenomena and facilitating the translation of computational results into practical applications.

Numerous in vitro validations utilizing cell lines have significantly contributed to our understanding of the molecular functions and regulatory roles of HDACs. These investigations typically involve the manipulation of HDAC expression levels or the application of HDAC inhibitors to cultured cell lines representing a spectrum of tissues and disease contexts [255,324,325]. Techniques such as immunoblotting, chromatin immunoprecipitation assays, and quantitative PCR analyses are commonly employed to dissect the intricate mechanisms underlying HDAC-mediated transcriptional regulation, cell cycle control, apoptosis, and cellular differentiation [113,326]. Moreover, cellular models have been instrumental in elucidating the crosstalk between HDACs and other signaling pathways, shedding light on their involvement in various physiological and pathological processes [113,327]. Additionally, the development and optimization of cell-based assays have facilitated the screening and validation of novel HDAC inhibitors, guiding the identification of promising candidates for therapeutic intervention [324,328].

One obstacle in identifying isoform-selective inhibitors stems from the limitations in current compound-screening technologies. The most commonly used in vitro deacetylation assay relies on monitoring the fluorescent signal resulting from HDAC-mediated degradation of the Fluor-De-Lys peptide substrate (Enzo Life Sciences). While this assay is robust and straightforward, screening for selectivity typically involves using purified, recombinant HDAC isoforms from baculovirus overexpression systems [329]. However, testing mammalian-cell derived HDAC proteins via immunoprecipitation of overexpressed isoforms from cell extracts introduces a typical error rate ranging from 30% to 60% [329]. Due to the significant error and labor-intensive nature of immunoprecipitation, many selective compounds have been exclusively tested against baculovirus-derived isoforms, such as tubastatin [330]. Yet, discrepancies arise when comparing inhibition data obtained from baculovirus- and mammalian cell–derived HDAC isoforms. For instance, apicidin exhibited potent activity against mammalian cell–derived HDAC1 (IC_50_ = 23 nM) [331], yet displayed variable potency against baculovirus-expressed HDAC1 (IC_50_ >10,000 nM or 0.7 nM in different reports) [329,332]. Although technical differences may account for these disparities, the similar assay formats suggest that the source of the HDAC isoforms can also impact screening results. Addressing this issue, Padige et al. introduced an enzyme-linked immunosorbent assay (ELISA)–based HDAC activity assay, aimed at utilizing mammalian cell–derived HDAC isoforms. Screening several known HDACis with diverse selectivity profiles validated the utility of this assay for inhibitor screening, suggesting its potential as a valuable tool for characterizing isoform-selective HDAC inhibitors against mammalian cell–derived HDAC isoforms [333].

Overall, the wealth of information garnered from in vitro studies using cell lines has been instrumental in advancing our knowledge of HDAC biology and in informing the development of targeted therapeutic strategies for a wide array of diseases.

## 6. Conclusions

In conclusion, undertaking a thorough examination of the conformational relationship of HDACis will facilitate the rational design of drugs and the development of effective and innovative treatments. Approved HDACis demonstrate efficacy against hematological malignancies, while numerous HDACis under evaluation show promise in various stages of clinical trials. In this scenario, computational techniques, including structure- and ligand-based virtual screening, pharmacophore modeling 3D-QSAR, molecular docking, dynamics simulations, and MM-PBSA/MM-GBSA ligand binding affinity calculations, have promoted and favoured the design of HDACis, by improving potency and/or selectivity. In particular, in this review it has been highlighted that combined computational strategies, through the application of multiple tools/force fields or scoring functions, stimulated the discovery of new lead compounds to optimize, leading to promising anticancer drugs within few years.

Although HDACis are still in the early stages of study, they have the potential to revolutionize our approach in fighting tumors, offering an attractive avenue for treating this complex pathology. Through the continued exploration of HDAC inhibition using computational methods, we establish the groundwork for revolutionary strides in cancer treatments, emphasizing the profound clinical and societal impact inherent in this field of inquiry.

## Figures and Tables

**Table 1:** Diseases associated with each HDAC isoform.

Class I		Zn^2+^-Dependent
**Isoform**	**Disease**	
HDAC1, HDAC2, HDAC3, HDAC8	**Cancer** (prostate, gastric, colorectal, Hodgkin lymphoma, lung, liver, acute lymphoblastic leukemia, breast, neuroblastoma); **Neurological diseases** (Huntington’s disease, Amyotrophic Lateral Sclerosis, Alzheimer’s Disease); **Metabolic diseases** (Diabetes, obesity); **Cardiovascular diseases**	
**Class II**		**Zn^2+^-Dependent**
**Isoform**	**Disease**	
HDAC4, HDAC5, HDAC6, HDAC7, HDAC9, HDAC10	**Cancer** (esophageal, colon, Hodgkin lymphoma, lung, acute lymphoblastic leukemia, breast, medulloblastoma); **Neurological diseases** (Huntington’s disease, Parkinson’s disease, Amyotrophic Lateral Sclerosis, Alzheimer’s Disease); **Cardiovascular diseases**	
**Class IV**		**Zn^2+^-Dependent**
**Isoform**	**Disease**	
HDAC11	**Cancer** (breast, renal, liver); **Neurological diseases** (Multiple Sclerosis); **Cardiovascular diseases**	
**Class III**		**NAD+-dependent**
**Isoform**	**Disease**	
SIRT1, SIRT2, SIRT3, SIRT4, SIRT5, SIRT6, SIRT7	**Cancer** (breast, pancreatic, colon, glioma); **Neurological disease** (Multiple Sclerosis); **Metabolic diseases** (Diabetes, obesity); **Cardiovascular diseases**	

**Table 2:** Description of the intricate role of each HDAC isoform in cancer biology and their distinct expression patterns across diverse tumor types.

Class I		Zn^2+^-Dependent
Isoform	Role in Cancer Biology	Expression in Cancer
HDAC1	(−) Apoptosis, (+) proliferation	Overexpressed in prostate (hormone-refractory), gastric ^A^, colorectal, breast ^B^, Hodgkin lymphoma, lung ^A^, and liver ^A^ cancer
HDAC2	(−) Apoptosis, (+) proliferation, (+) aneuploidy	Overexpressed in colorectal ^A^, gastric ^A^, prostvate ^A^, Hodgkin lymphoma, acute lymphoblastic leukemia
HDAC3	(−) Differentation, (+) proliferation	Overexpressed in lung, gastric ^A^, breast ^AB^, Hodgkin lymphoma, acute lymphoblastic leukemia Downregulated in liver cancer
HDAC8	(−) Differentation, (+) proliferation	Overexpressed in neuroblastoma
**Class II**		**Zn^2+^-Dependent**
**Isoform**	**Role in Cancer Biology**	**Expression in Cancer**
HDAC4	(−) Differentation, (+) Angiogenesis	Overexpressed in esophageal cancer
HDAC5	(−) Differentation, (−) Migration	Overexpressed in medulloblastoma Downregulated in lung, colon cancer and acute myeloid leukaemia
HDAC6	(+) Migration	Overexpressed in breast cancer Downregulated in lung cancer
HDAC7	(+) Angiogenesis	Overexpressed in acute lymphoblastic leukemia Downregulated in lung cancer
HDAC9	(+) Angiogenesis	Overexpressed in medulloblastoma
HDAC10	(+) Angiogenesis	Overexpressed in lung cancer
**Class IV**		**Zn^2+^-Dependent**
**Isoform**	**Role in Cancer Biology**	**Expression in Cancer**
HDAC11		Overexpressed in breast, renal and liver cancer

(+) Increased activity; (−) Reduced activity; ^A^ Independent prognosis indicator; ^B^ Associated with enhanced prognosis.

**Table 3:** Size (number of amino acids), residues involved in the Zn^2+^ coordination, cellular distribution, corresponding complex and chromosome location for each HDAC isoform [94,95,96,97,98].

Class I			Zn^2+^-Dependent		
**Isoform**	**Size (aa)**	**Zn Coordination**	**Cellular Distribution**	**Complex**	**Chromosome** **Location**
HDAC1	482	His140 His141 Asp176 Asp264	Nuclear	Sin3, NURD	1p34
HDAC2	488	His141 His142 Asp177 Asp265	Nuclear	Sin3, NURD	6q21
HDAC3	428	His134 His135 Asp170 Asp259	Nuclear	NCOR1/NCOR2-GPS2-TBL1X	5q31
HDAC8	377	His142 His143 Asp178 Asp267	Nuclear		Xq13
**Class II**			**Zn^2+^-Dependent**		
**Isoform**	**Size (aa)**	**Zn Coordination**	**Cellular Distribution**	**Complex**	**Chromosome** **Location**
HDAC4	1084	His802 His803 Asp840 Asp934	Nuclear, cytoplasm	NCOR1/NCOR2	q37.2
HDAC5	1122		Nuclear, cytoplasm		17q21
HDAC6	1215	H610 H611 Asp649 Asp742	Nuclear, cytoplasm		Xp11.22–23
HDAC7	952	His669 His670 Asp707 Asp801	Nuclear, cytoplasm	NCOR1/NCOR2	12q13.1
HDAC9	1011		Nuclear, cytoplasm		p21–p15
HDAC10	669		Nuclear, cytoplasm	NCOR2	22q13.31
**Class IV**			**Zn^2+^-Dependent**		
**Isoform**	**Size (aa)**	**Zn Coordination**	**Cellular Distribution**	**Complex**	**Chromosome** **Location**
HDAC11	347		Nuclear	-	-
**Class III (SIRT)**			**NAD+-Dependent**		
**Isoform**	**Size (aa)**		**Cellular Distribution**	**Complex**	**Chromosome** **Location**
SIRT1	747		Nuclear, cytoplasm	eNoSC	
SIRT2	389		Nuclear, cytoplasm		
SIRT3	399		Mitochondria	FoxO3A	
SIRT4	314		Mitochondria		
SIRT5	310		Nuclear, cytoplasm, mitochondria		6p23
SIRT6	355		Nucleas, endoplasmic reticulum		
SIRT7	400		Nuclear, cytoplasm		17q25.3

**Table 4:** Omics system technologies used to analyze HDACs and HDACis.

Omics	Analysis	Detecting
**Chemoproteome**	MS, beads MS, MudPIT	Protein/HDACi interaction
**Epigenome**	ChIP-seq, ChIP-qPCR, ChIP-chip, DNase-seq, MNase-seq, ATAC-seq, MBD-seq, RNA-seq, NA-seq, HT-FAIRE	Histone modification and chromatin accessibility
**Acetylome**		Protein modification
**Transcriptome**	Microarray, miRNA microarray, miRNA-seq, mRNA-seq, splicing-sensitive microarray, TempO-seq, GRO-seq, ChIP-seq	Gene expression
**Proteome**	LC-MS/MS, SILAC, HSMS, MS acetylome	Protein expression
**Metabolome**	MS metabolomics, NMR, LC/GC-MS/MS	Metabolic physiology

MS (mass spectrometry); MudPIT (Multidimensional protein identification technology); ChIP (Chromatin immunoprecipitation); MNase (micrococcal nuclease); ATAC (assay for transposase-accessible chromatin); MBD (Methyl-CpG Binding Domain); FAIRE (formaldehyde-assisted isolation of regulatory elements); TempO (templated oligo assay); GRO (Global run-on); LC-MS/MS (liquid chromatography–tandem mass spectrometry); SILAC (Stable isotope labeling by amino acids in cell culture); HSMS (High-Resolution Mass Spectrometry); NMR (nuclear magnetic resonance); GC (gas chromatography) [116].

**Table 5:** A 2D chemical structure, related disease and selectivity of current HDAC inhibitors divided by chemical class (hydroxamic acids, benzamides, short-chain fatty acids, and cyclic tetrapeptides).

Class	Inhibitor	2D Structures	Selectivity	Disease
Hydroxamic acids	Vorinostat	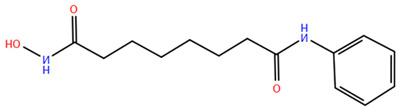	Pan	Cutaneous T-cell lymphoma (Approved) Alzheimer’s disease (Phase I)
	Trichostatin A	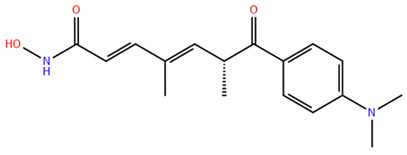	Pan	Preclinical use
	Belinostat	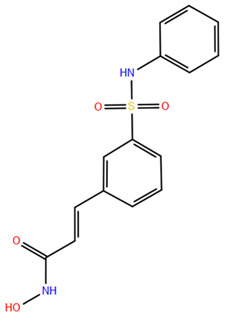	Pan	Peripheral T-cell lymphoma (Approved)
	Panobinostat	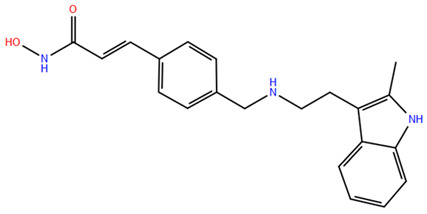	Pan	Multiple myeloma (Approved)
	Givinostat	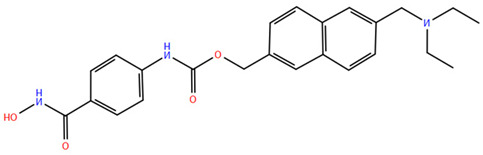	Pan	Relapsed leukemia Multiple myeloma (Phase II)
	Resminostat	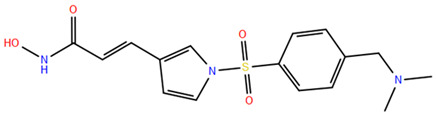	Pan	Hepatocellular carcinoma (Phase I and II)
	Abexinostat	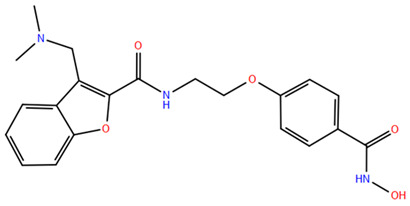	Pan	B-cell lymphoma (Phase II)
	Quisinostat	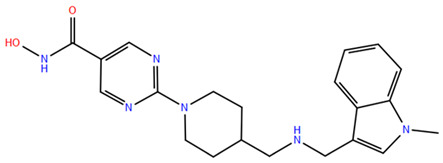	Pan	Multiple myeloma (Phase I)
	Rocilinostat	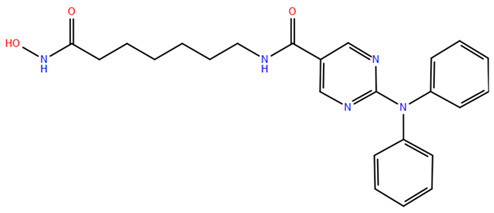	II	Multiple myeloma (Phase I)
	Pracinostat	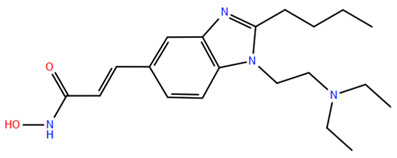	I, II, IV	Prostate cancer (Phase II)
	CHR-3996	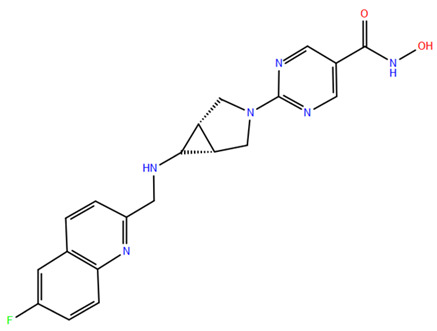	I	Advanced/metastatic solid tumors refractory to standard therapy (Phase I)
Benzamides	Entinostat	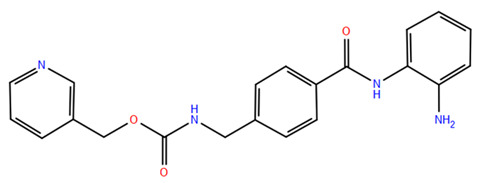	I	Breast cancer, Hodgkin’s lymphoma, non-small cell lung cancer (Phase II and Phase III)
	Tacedinaline	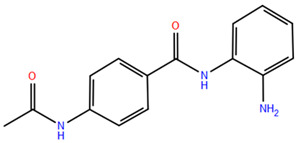	I	Hormone receptor-positive breast cancer, Non-small cell lung cancer and pancreatic cancer (Phase III)
	4SC202	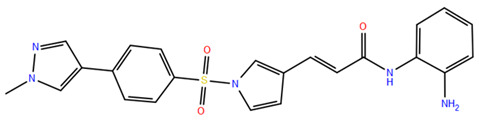	I	Advanced hematological malignancies (Phase I)
	Mocetinostat	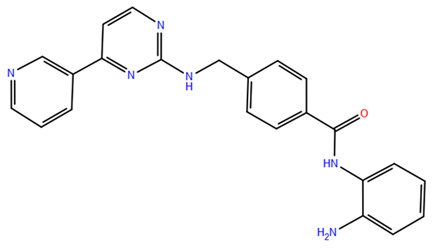	I, IV	Hodgkin’s lymphoma (Phase II)
Short-chain fatty acids	Valproic acid	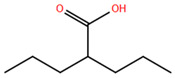	I, IIa	Epilepsia, bipolar disorders, and migraine (Approved)
	Butyric acid	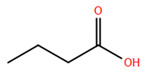	I, II	Multiple conditions (Phase II)
	Phenylbutyric acid	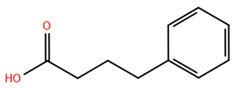	I, II	Multiple conditions (Phase I)
Cyclic tetrapeptides	Romidepsin	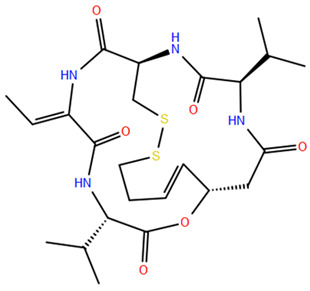	I	Cutaneous T-cell lymphoma (Approved)
