# Correction: Curcio et al. The Histone Deacetylase Family: Structural Features and Application of Combined Computational Methods. *Pharmaceuticals* 2024, *17*, 620

**DOI:** 10.3390/ph17111520

**Published:** 2024-11-12

**Authors:** Antonio Curcio, Roberta Rocca, Stefano Alcaro, Anna Artese

**Affiliations:** 1 Dipartimento di Scienze della Salute, Campus “S. Venuta”, Università degli Studi “Magna Græcia” di Catanzaro, Viale Europa, 88100 Catanzaro, Italy; antonio.curcio@unicz.it (A.C.); alcaro@unicz.it (S.A.); artese@unicz.it (A.A.); 2 Net4Science S.r.l., Università degli Studi “Magna Græcia” di Catanzaro, Viale Europa, 88100 Catanzaro, Italy

## Missing Citation

In the original publication [1], references 11, 19 and 173 [2,3,4] were not cited. The citations have now been inserted in Section 1.1. Classification of HDAC Family (Paragraphs 1 and 2) and Section 2.4. Future Perspectives and PROTACs (Paragraph 2). They should read:

### 1.1. Classification of HDAC Family

As reported in the recent review of Han and co-workers [11], so far, according to their 3D structure, function, and sequence homology, 18 isoforms of human HDACs have been identified.

Class IIa HDACs possess a single catalytic domain, whereas Class IIb members have two catalytic domains [18]. HDAC4 and HDAC5, belonging to Class IIa, are found in the brain, heart, and skeletal muscle [19]. In contrast, HDAC7 is mainly expressed in the heart, lung, placenta, pancreas, skeletal muscle, and thymus [20].

### 2.4. Future Perspectives and PROTACs

PROTACs have been shown to selectively degrade specific HDACs and other proteins, which could enhance the effectiveness of cancer immunotherapies. However, creating effective HDAC-PROTACs is complex due to factors like linker length, E3 ligase selection, and cell type specificity. A more detailed discussion on the development of HDAC-PROTACs is available in the review by Patel et al. [173].

## Text Correction

There were some errors in the original publication. A certain similarity to other published works was found.

A correction has been made to the Abstract, where the sentences “Computational methods have aided the discovery of HDAC inhibitors with the desired potency and/or selectivity. These methods include ligand-based approaches, such as scaffold hopping, pharmacophore modeling, three-dimensional quantitative structure–activity relationships, and structure-based virtual screening (molecular docking). Moreover, recent developments in the field of molecular dynamics simulations, combined with Poisson–Boltzmann/molecular mechanics generalized Born surface area techniques, have improved the prediction of ligand binding affinity.” were changed to “Computational techniques have greatly facilitated the discovery of HDAC inhibitors that achieve the desired potency and selectivity. These techniques encompass ligand-based strategies such as scaffold hopping, pharmacophore modeling, three-dimensional quantitative structure–activity relationships (3D-QSAR), and structure-based virtual screening (molecular docking). Additionally, advancements in molecular dynamics simulations, along with Poisson–Boltzmann/molecular mechanics generalized Born surface area (PB/MM-GBSA) methods, have enhanced the accuracy of predicting ligand binding affinity.”

Some corrections have been made to Section 1. Introduction, where the sentences “Histone deacetylases (HDACs), also known as lysine deacetylases (KDACs), are zinc (Zn^2+^)-dependent or nicotinamide adenine dinucleotide (NAD^+^)-dependent proteolytic enzymes. They are involved in transcriptional repression and chromatin condensation mechanisms through controlling the acetylation state of lysine side chains in histone tails” were changed to “Histone deacetylases (HDACs), also known as lysine deacetylases (KDACs), are proteolytic enzymes that depend on either zinc (Zn^2+^) or nicotinamide adenine dinucleotide (NAD^+^). They play a crucial role in transcriptional repression and chromatin condensation by regulating the acetylation state of lysine residues on histone tails”; the sentence “Specifically, the HDAC protein family normally removes the acetate moiety from acetylated ε-amino groups of histone lysine and other non-histone proteins” was changed to “HDACs achieve this by removing the acetate moiety from acetylated ε-amino groups on histone lysines and other non-histone proteins”; the sentence “Moreover, HDACs can indirectly regulate other post-translational modifications (PTMs) by releasing the acetyl group from lysine so that other PTMs, for instance ubiquitination, can mark on the loci” was changed to “Furthermore, HDACs can indirectly influence other post-translational modifications (PTMs) by removing the acetyl group from lysine residues, thereby allowing other changes, such as ubiquitination, to occur at those sites”; the sentence “The enzymatic activities of HDAC isoforms are responsible for the maintenance of several normal physiological processes, such as cell proliferation, apoptosis, neurogenesis, and epigenetic regulations” was changed to “The enzymatic activities of HDAC isoforms are crucial for maintaining various normal physiological processes, including cell proliferation, apoptosis, neurogenesis, and epigenetic regulation”; and the sentence “Specifically, irregular HDAC expression and its abnormal deacetylation activity have become a very popular and interesting topic for the prevention of cancer generation and progression” was changed to “Specifically, abnormal HDAC expression and deacetylation activity have become a major focus in the study of cancer prevention and progression”.

Some corrections have been made to Section 1.1. Classification of HDAC Family, where the sentences “The Class III subfamily is known as Sirtuins, due to its strong resemblance to the yeast Sir2 protein. It contains seven members (SIRT-7) and uses NAD^+^ for its ADP-ribosyltransferase and histone deacetylase enzymatic activities [23]” were changed to “The Class III subfamily, known as Sirtuins, comprises seven members (SIRT1-7). Named for their resemblance to the yeast Sir2 protein, Sirtuins utilize NAD^+^ to carry out their ADP-ribosyltransferase and histone deacetylase activities [25]”.

Some corrections have been made to Section 1.2. HDAC Structure and Function, where the sentences “HDACs typically include a core structural domain known as the HDAC structural domain, which consists of two highly conserved isoforms: the HDAC N-terminal structural domain and the HDAC central structural domain [6]. Within the HDAC structural domain, some catalytic sites, such as zinc ions and arginine residues, are crucial for their catalytic activity [31]. On the other hand, the HDAC C-terminal structural domain represents a more miscellaneous region, characterized by variations in length and amino acid sequences among different HDAC types [32]. Moreover, HDACs can form complexes with various proteins, including cell cycle regulatory proteins and transcription factors [33]” were changed to “HDACs generally possess a core structural domain called the HDAC structural domain, which includes two highly conserved regions: the HDAC N-terminal and the HDAC central structural domains [6]. These domains feature critical catalytic sites, such as zinc ions and arginine residues, essential for their enzymatic activity [33]. In contrast, the HDAC C-terminal domain is more variable, with differences in length and amino acid sequences across different HDAC types [34]. Additionally, HDACs can interact with various proteins, including those involved in cell cycle regulation and transcription factors [35]”; the sentences “The earliest identified members of the Class I HDAC family include Rpd3 in budding yeast, along with HDACs1–3 and 8 all tumbling into this class. Their N-terminus contains the catalytic structural domains and exhibits a sequence conservation of 40–70% with the catalytic domain of yeast Rpd3 [34]” were changed to “Among the earliest discovered members of the Class I HDAC family was Rpd3 from budding yeast, along with human HDACs 1, 2, 3, and 8. These proteins are categorized into Class I due to their shared structural characteristics. The N-terminal regions of these HDACs contain the catalytic domains, which are essential for their enzymatic activity. Notably, the sequences of these catalytic domains exhibit a substantial degree of conservation, ranging between 40% and 70%, when compared to the catalytic domain found in yeast Rpd3 [36]”; the sentences “HDAC1 and HDAC2 are exclusively localized in the nucleus, while HDAC3 contains a nuclear localization signal (NLS) region and a nuclear export signal (NES) region. Its localization may vary depending on cell type and environmental conditions [37]” were changed to “HDAC1 and HDAC2 are strictly confined to the nucleus. On the other hand, HDAC3 features both a nuclear localization signal (NLS) and a nuclear export signal (NES), which means its presence within the nucleus can be dynamic. The localization of HDAC3 is influenced by various factors, including the specific cell type and the surrounding environmental conditions [39]”; the sentences “This framework consists of a core featuring eight parallel β-fold bundles forming a β-fold sheet, surrounded by more than thirteen α-helices and elongated loops stemming from the C-terminus of the β-fold, thus creating a narrow hydrophobic channel [4]. Within HDAC8, the hydrophobic channel is delineated by specific residues, including Phe152, Phe208, His180, Gly151, Met274, and Tyr306. Contrastingly, in other Class I subfamily members (HDAC1-3), most residues within this channel are conserved, except for Met274, which is substituted by leucine residues [39]” were changed to “The enzyme’s structure is anchored by a core that includes eight parallel β-strands organized into a β-sheet, which is flanked by over thirteen α-helices and extended loops expanding from the C-terminus of the β-sheet. This arrangement creates a narrow hydrophobic channel [4]. Within HDAC8, this channel is specifically outlined by residues such as Phe152, Phe208, His180, Gly151, Met274, and Tyr306. However, in other members of the Class I HDAC subfamily (HDAC1-3), most of these residues are conserved, with the exception of Met274, which is replaced by leucine residues [41]”; the sentences “At the bottom of the channel, the Zn^2+^ ion forms a chelate involving His180, Asp178, and Asp267 of HDAC, the acetyl group oxygen of the substrate, and the water molecule oxygen participating in the hydrolysis reaction. Aside from the substrate binding site and the zinc ion binding site, two metal ion binding sites are found in the catalytic domain of HDAC (Site 1 and Site 2) [40]” were changed to “At the bottom of the channel, the Zn^2+^ ion forms a chelate complex with the His180, Asp178, and Asp267 of the HDAC, the oxygen atom from the substrate’s acetyl group, and a water molecule participating in the hydrolysis process. Besides the substrate binding site and the zinc ion binding site, the catalytic domain of the HDAC also features two additional metal ion binding sites, known as Site 1 and Site 2 [42]”; the sentences “α-helix H1 in HDAC8 adopts a conventional α-helix structure, while loop L1 is two amino acid residues shorter than that of HDAC3. Additionally, loop L6 in HDAC8 contains a proline residue, causing the loop to be slightly distant from the catalytic site. As a result, HDAC8 maintains a relatively open catalytic pocket, which could potentially facilitate the access of substrate to its catalytic core. These structural differences make HDAC8 more compatible with its catalytic core and promote easier substrate access [43]. The elucidation of HDAC8’s crystal structure is crucial for understanding the structural dynamics and catalytic mechanisms of zinc ion-dependent HDACs, particularly within the Class I subgroup (Figure 3) [43]” were changed to “in HDAC8, α-helix H1 assumes a standard α-helix conformation, whereas loop L1 is two amino acids shorter compared to HDAC3. Moreover, loop L6 in HDAC8 includes a proline residue, which positions the loop slightly farther from the catalytic site. This results in a more open catalytic pocket in HDAC8, potentially improving the substrate’s access to the catalytic core. These structural distinctions enhance the alignment of HDAC8’s catalytic core and facilitate more efficient substrate binding [45]. Understanding the crystal structure of HDAC8 is pivotal for gaining insights into the structural dynamics and catalytic processes of zinc-dependent HDACs, particularly those in the Class I subgroup (Figure 3) [45]”; the sentences “Enzymes belonging to Class II are further divided into two categories: Type a and Type b. The first Type encompasses HDAC4, 5, 7, and 9, while Type b comprises HDAC6 and HDAC10, which were recently discovered [46] (Figure 4). HDACs of Class IIa share a common catalytic core structural domain located at their C-terminus. They also have a unique and conserved N-terminal extension region, which contains multiple binding sites” were changed to “Class II enzymes are categorized into Type a and Type b. Type a HDACs include HDAC4, 5, 7, and 9, whereas Type b consists of the recently discovered HDAC6 and HDAC10 [48] (Figure 4). Class IIa HDACs share a common catalytic core domain at their C-terminus and feature a distinctive, conserved N-terminal extension with multiple binding sites”; the sentences “By regulating microtubule assembly and the localization of microtubule motor complexes, HDAC6 influences microfilament-based cell motility and the interaction of cortical actin with microfilaments [56]. Thus, its inhibition results in the hyperacetylation of microtubule proteins, enhancing intracellular vesicular transport, a process associated with neurological disorders like Parkinson’s and Huntington’s disease [57]” were changed to “HDAC6 regulates microtubule assembly and the positioning of microtubule motor complexes, affecting cell motility and the interaction of cortical actin with microfilaments [58]. Therefore, the inhibition of HDAC6 leads to the hyperacetylation of microtubule proteins, which improves intracellular vesicular transport and is linked to neurological disorders such as Parkinson’s and Huntington’s disease [59]”; the sentences “It harbors two conserved deacetylation catalytic domains at the N-terminal end. While the C-terminal region shares sequence similarity with the N-terminal end, it lacks deacetylase activity” were changed to “It contains two conserved deacetylation catalytic domains at the N-terminal end. Although the C-terminal region resembles the N-terminal sequence, it does not possess deacetylase activity. The cytoplasmic localization is determined by a leucine-rich domain at the C-terminal end”; the sentences “Specifically, their catalytic structural domain shows an elliptical shape and comprises two large and two small structural domains, each encompassing approximately 270 amino acid residues. The relatively conserved large structural domain adopts a typical Rossmann fold structure, featuring a central β-sheet and six β-strands” were changed to “The catalytic domain of these proteins shows an elliptical configuration and is organized into two prominent and two smaller structural segments, each spanning approximately 270 amino acids. The more conserved of the two larger segments features a Rossmann fold architecture, which includes a central β-sheet surrounded by six β-strands”; the sentence “The zinc finger structural domain is made up of three reverse parallel β-strands and one α-helix [68]” was changed to “The zinc finger domain consists of three β-strands arranged in a reverse parallel pattern along with a single α-helix [70]”; the sentence “Notably, the largest loop, i.e., a β1-α2 loop or cofactor binding loop, is a part of the NAD^+^ binding site and has a highly dynamic structure, which is critical for catalytic reactions” was changed to “Of particular importance is the largest loop, known as the β1-α2 loop or cofactor binding loop. This loop, integral to the NAD^+^ binding site, exhibits significant structural flexibility, which is crucial for the enzyme’s catalytic activity”; the sentences “As researchers delve deeper into the structural complexities of HDACs, they are gradually uncovering distinctions among various subgroups and subtypes. This knowledge offers a rational explanation for the selectivity of some HDAC inhibitors. For example, a series of novel o-phenylenediamine HDAC inhibitors were found to selectively inhibit HDAC1 and HDAC2 [90]. After conducting a detailed analysis of homologously modeled HDAC1 and HDAC3, researchers have discovered a structural difference in an amino acid located in the cavity at the bottom of the catalytically active center. This difference involves the substitution of the Ser113 residue of HDAC1 with the Tyr96 residue of HDAC3, which may serve as the structural basis for selectivity. Additionally, the lack of isoform selectivity in most current HDAC inhibitors is due to the highly conserved amino acid sequence of the catalytic active center of Zn^2+^-dependent HDAC [91]” were changed to “As research into the structural complexities of HDACs advances, distinct differences among various subgroups and subtypes are being revealed. This understanding helps explain the selectivity of certain HDAC inhibitors. For instance, novel o-phenylenediamine HDAC inhibitors have been shown to specifically target HDAC1 and HDAC2 [92]. The analysis of HDAC1 and HDAC3 structures revealed a key difference: a substitution of Ser113 in HDAC1 with Tyr96 in HDAC3 at the bottom of the catalytic center. This variation likely contributes to the observed selectivity. However, most current HDAC inhibitors lack isoform selectivity due to the highly conserved amino acid sequence in the catalytic center of Zn^2+^-dependent HDACs [93]”; and the sentences “To develop inhibitors selective toward specific subtypes, researchers are employing a successful strategy that takes into account the structural differences around the entrance to the active center of each HDAC8 subtype. A comprehensive understanding of the structural differences of each HDAC isoform will also be invaluable in creating potent and specific inhibitors” were changed to “Researchers are employing an effective strategy to develop inhibitors that specifically target individual HDAC8 subtypes by considering the structural variations around the entrance to each subtype’s active site. A thorough grasp of the structural differences among HDAC isoforms will be crucial for developing potent and specific inhibitors”.

Some corrections have been made to Section 2. HDAC Inhibitors, where the sentences “However, they face several challenges, such as low bioavailability, rapid metabolism, irreversible differentiation, and lack of selectivity towards cancer cells. Therefore, it is crucial to explore the various functions of different HDACs to develop more effective and selective HDAC inhibitors.” were changed to “However, they face several challenges, including poor bioavailability, rapid metabolic breakdown, irreversible differentiation effects, and insufficient selectivity for cancer cells. To address these issues, it is essential to investigate the diverse functions of different HDACs in order to create more effective and targeted HDAC inhibitors”; the sentence “For cell death in many cancer cells, HDACis can activate either the extrinsic pathway, influencing the receptor death pathway, or the intrinsic pathway, affecting the mitochondrial pathway [127].” was changed to “In many cancer cells, HDAC inhibitors can induce cell death by activating either the extrinsic pathway, which affects receptor-mediated apoptosis, or the intrinsic pathway, which involves mitochondrial signaling [129].”; the sentences “This model comprises three essential elements:

(1) the cap structure (Surface Recognition Domain), which typically constitutes a hydrophobic aromatic moiety that interfaces with the enzyme surface;
(2) a Zn^2+^ binding group (ZBG), such as isohydroxamic acid, carboxylic acid, or benzamide, coordinating the Zn^2+^ ion at the enzyme catalytic center;
(3) a Linker, which may be a saturated or unsaturated linear chain or a hydrophobic long chain with a ring structure, connecting the cap structure to the ZBG [133,134].

Studies on co-crystalized complexes of isohydroxamic acid HDACis and HDACs have elucidated the interaction between the cap structure and the amino acids proximal to the enzyme catalytic site. Meanwhile, the ZBG structure binds to the metal ion at the bottom of the active site to form the complex [45].”

were changed to “This model comprises three essential elements:

(1) the cap structure (Surface Recognition Domain): this component generally features a hydrophobic aromatic group that interacts with the enzyme surface;
(2) Zn^2+^-binding group (ZBG): this group, which can include compounds such as isohydroxamic acid, carboxylic acid, or benzamide, binds to the Zn^2+^ ion at the enzyme’s catalytic site;
(3) linker: this element, which can be a linear chain (either saturated or unsaturated) or a hydrophobic long chain with a ring structure, connects the cap structure to the ZBG [135,136].

Studies of co-crystalized complexes between isohydroxamic acid HDAC inhibitors and HDACs have clarified how the cap structure interacts with amino acids near the enzyme’s catalytic site. Additionally, the zinc-binding group (ZBG) structure binds to the metal ion at the bottom of the active site, forming a stable complex [47].” The sentences “The long linker chain interacts with amino acid residues, occupying the active site, through forces like van der Waals interactions. On the other hand, the cap structure functions as a cover for the entrance to the enzyme active site [45]. HDACis work by competitively inhibiting the binding of acetyl-lysine residues of the substrate to the enzyme active site.” were changed to “The extended linker chain engages with residues within the active site through van der Waals interactions, while the cap structure acts as a barrier, blocking access to the enzyme’s active site entrance [47]. HDACis work by competing with acetyl-lysine residues for binding to the enzyme’s active site, thereby blocking their interaction and disrupting the enzyme’s activity.”; the sentence “A comparative analysis of clinical HDACis SAHA, entinostat (MS275), and valproic acid, utilizing isohydroxamic acid, benzamide, and carboxylic acid as chelating groups, respectively, underscores significant differences in their inhibitory activity against HDACs.” was changed to “A comparative analysis of clinically used HDACis, including SAHA, entinostat (MS275), and valproic acid—each with distinct chelating groups—reveals notable differences in their effectiveness against HDACs.”; and the sentence “Moreover, the most effective enzyme inhibitory activity was observed when the carbon number of the linker region (n) is six.” was changed to “Moreover, the highest level of enzyme inhibition was achieved when the linker region (n) contained six carbon atoms.”

Some corrections have been made to Section 2.1. Isohydroxamic Acids, where the sentences “Furthermore, isohydroxamic acid is susceptible to hydrolysis and glucuronidation, resulting in unfavorable pharmacokinetic characteristics and reduced in vivo efficacy [142]. Regarding the linker domain of isohydroxamic acid, either linear or cyclic structures, as well as saturated or unsaturated configurations, can be observed. Regarding the linear, straight-chain linker isohydroxamic acid HDAC inhibitors, the cap structure plays a pivotal role in compound modification and optimization, resulting in a diverse range of HDAC inhibitors.” were changed to “Furthermore, hydrolysis and glucuronidation can adversely affect isohydroxamic acid by impairing its pharmacokinetic properties and reducing its effectiveness in biological systems [144]. Additionally, the linker domain of isohydroxamic acid can vary, featuring either linear or cyclic structures and saturated or unsaturated configurations.”; the sentence “Due to this, in designing HDAC inhibitors, more complex cap structures such as branching caps are often employed.” was changed to “Due to this, in the development of HDACis, more complex cap structures, such as branching caps, are commonly employed.”; the sentences “This compound has shown potential in treating hematologic cancers, with a different efficacy in B-cell lymphomas, such as diffuse large B-cell lymphoma, follicular lymphoma and mantle cell lymphoma. For solid tumors like prostate and pancreatic cancers, SAHA has demonstrated the inhibition of the Akt/FOXO3a signaling pathway, which stimulates apoptosis in prostate tumor cells [145]. Additionally, SAHA plays a crucial role in inducing autophagy in tumor cells and preventing acute graft-versus-host disease. However, it is associated with significant toxicity, including fatigue, diarrhea, anorexia, bone marrow suppression and thrombocytopenia, due to its broad-spectrum inhibitory capacity [146]. Recent research has shown a promising potential in enhancing the selectivity of HDAC inhibitors, or developing new ones based on SAHA’s fundamental pharmacodynamic moiety. For instance, by replacing the hydrogen atom (H) at the C2 position of SAHA’s hydrophobic long chain with aliphatic or aromatic hydrocarbons, a new analog named C2-R-SAHA was created. This analog has shown the ability to enhance its selectivity for HDAC6 and 8 [147]. Thanks to the molecular docking approach, highly conserved active catalytic regions across all HDAC isoforms were observed, with class I HDAC exhibiting a narrower hydrophobic channel than HDAC6. Thus, by substituting aliphatic hydrocarbons on the hydrophobic chain of SAHA, it is possible to increase the barrier to the catalytic channel, which in turn impedes the catalysis of compounds HDAC1, 2 and 3. Similarly, by replacing unsaturated hydrocarbons on aromatic, cyclic, or adjacent isohydroxamic acid groups, it is possible to enhance the selectivity of HDAC6, as observed in tubastatin A [148].” were changed to “This compound has shown promise in treating hematologic cancers, including diffuse large B-cell lymphoma, follicular lymphoma, and mantle cell lymphoma. In solid tumors such as prostate and pancreatic cancers, SAHA inhibits the Akt/FOXO3a signaling pathway, promoting apoptosis in prostate cancer cells [147]. SAHA also induces autophagy in tumor cells and helps prevent acute graft-versus-host disease. However, its broad-spectrum inhibition leads to significant side effects, including fatigue, diarrhea, anorexia, bone marrow suppression, and thrombocytopenia [148]. Recent research aims to improve the selectivity of HDAC inhibitors or develop new ones based on SAHA’s core pharmacodynamic structure. For example, a new analog, C2-R-SAHA, was created by replacing the hydrogen atom at the C2 position of SAHA’s hydrophobic long chain with aliphatic or aromatic hydrocarbons. This analog has shown the ability to enhance its selectivity for HDAC6 and 8 [149]. The molecular docking analysis identified that Class I HDACs possess a more constricted hydrophobic channel compared to HDAC6. As a result, introducing aliphatic hydrocarbons into the hydrophobic chain of SAHA can enhance the obstruction within this catalytic channel, thereby reducing the effectiveness of HDAC1, 2, and 3. On the other hand, modifying the structure by incorporating aromatic, cyclic, or neighboring isohydroxamic acid groups can boost the selectivity for HDAC6, as exemplified by tubastatin A [150].”; and the sentences “This increased activity is largely attributed to the bridging region of TSA, which includes a diene and an R-type methyl group. However, researchers have determined that these features alone do not fully explain the potency of TSA. The arylamine ring in the surface recognition region may also play a significant role in its effectiveness by interacting with amino acid residues in the enzyme capsule [149].” were changed to “The enhanced activity of TSA is primarily due to its bridging region, which contains a diene and an R-type methyl group. However, this alone does not fully account for TSA’s potency. The arylamine ring in the surface recognition region likely contributes significantly to its effectiveness by interacting with amino acid residues within the enzyme’s active site [151].”

Some corrections have been made to Section 2.2. Benzamide Derivatives, where the sentence “Conversely, one side of the aromatic ring enters the catalytic “foot pocket,” prompting the repositioning of its residues to accommodate the aryl portion.” was changed to “Conversely, one portion of the aromatic ring fits into the catalytic “foot pocket,” leading to a rearrangement of residues to accommodate the aryl group.”

Some corrections have been made to Section 2.3. Cyclic Peptides, where the sentence “for interaction with Zn^2+^ and polar amino acid residues in the HDAC duct, and an epoxy group for alkylating the HDAC active site, thereby irreversibly inhibiting enzyme activity.” was changed to “for interacting with Zn^2+^ and polar amino acids within the HDAC channel, and an epoxy group to alkylate the HDAC active site, thereby causing irreversible enzyme inhibition.”; the sentences “In vivo, it necessitates hydrolysis to release the sulfhydryl moiety of the zinc-chelating group, enabling effective chelation with zinc metal ions for enzyme inhibition. The larger cap group enhances interaction with amino acids at the active pocket periphery, thereby increasing target affinity [156].” were changed to “In vivo, hydrolysis is needed to free the thiol component of the zinc-chelating group, allowing for effective zinc ion binding for enzyme inhibition. The larger cap group improves interaction with peripheral amino acids, thereby enhancing target affinity [158].”; the sentences “It derives from Gram-negative pigmented bacillus No. 968 and possesses a caged bicyclic phenolic peptide structure with rare disulfide bonds. These bonds are activated within human cells post-metabolism [158].” were changed to “It is derived from Gram-negative pigmented bacillus No. 968 and features a caged bicyclic phenolic peptide structure with uncommon disulfide bonds, which become activated in human cells following metabolism [160].”; and the sentence “Upon entering the cell, FK228 undergoes activation through glutathione reduction, allowing the Red-FK228 free sulfhydryl group to interact with the Zn^2+^ active site, thereby preventing HDAC from binding to the substrate.” was changed to “This process liberates the Red-FK228 free sulfhydryl group, which then interacts with the Zn^2+^ active site, inhibiting HDAC from binding to its substrate.”

A correction has been made to Section 2.4. Future Perspectives and PROTACs; the content after correction is as follows:

Despite the considerable progress made, numerous unresolved issues in the exploration of HDAC inhibitors (HDACis) remain. Primarily, the majority of HDACis currently available are broad-spectrum inhibitors. In fact, these compounds compete for Zn^2+^ within the enzyme active site, lacking specificity towards distinct isoforms. It is possible to achieve a degree of selectivity for HDAC isoforms by interrupting specific HDAC activities essential for protein–protein interactions [161].

HDAC1, 2, and 3 are components of multiprotein complexes found in the nucleus, and their displacement greatly reduces enzyme activity. Inositol phosphate, a key regulatory factor in these complexes, is essential for their formation and enzyme activation by interacting with arginine residues near the active site. Interrupting this interaction can enhance the inhibition of HDAC activity [162]. HDAC inhibitors commonly use groups such as isohydroxamic acid or carboxylic acid to bind Zn^2+^ in HDACs, but these groups can also target other metalloenzymes, leading to toxicity and limiting their clinical use [163]. To improve therapeutic outcomes and minimize side effects, combination therapies with HDAC inhibitors are being explored. HDAC-PROTACs represent a promising approach by selectively degrading HDACs (Figure 8), reducing off-target effects, and potentially overcoming drug resistance [164–172]. PROTACs have been shown to selectively degrade specific HDACs and other proteins, which could enhance the effectiveness of cancer immunotherapies. However, creating effective HDAC-PROTACs is complex due to factors like linker length, E3 ligase selection, and cell type specificity. A more detailed discussion on the development of HDAC-PROTACs is available in the review by Patel et al. [173]. Despite these challenges, HDAC-PROTACs hold significant promise for clinical applications, with ongoing research aimed at enhancing their selectivity and therapeutic potential in cancer and other diseases [174,175].

Some corrections have been made to Section 4.1. Molecular Modeling, where the sentence “and of a novel Aminotetralin Class of HDAC6 and HDAC8 selective inhibitors with potent inhibitory activity against neuroblastoma BE(2)C cells [217]” was changed to “this approach also led to the identification of a new class of Aminotetralin-based inhibitors selective for HDAC6 and HDAC8, demonstrating significant inhibitory effects on neuroblastoma BE(2)C cells [220]”; the sentences “a pair of HDAC6-selective inhibitors with 2-mercaptoquinazolinone as the cap moiety were drawn for the first time. This design foresaw manipulating the surface recognition group (quinazolinone core as a cap) and linker while retaining the hydroxamic acid side chains at the C-2 or N-3 position [218]” were changed to “two HDAC6-selective inhibitors were developed for the first time, incorporating 2-mercaptoquinazolinone as the cap moiety. This design strategy involved altering the surface recognition group by using the quinazolinone core as the cap and fine-tuning the linker, while maintaining hydroxamic acid side chains at either the C-2 or N-3 position [221]”; the sentences “the conventional pharmacophore of HDAC inhibitors comprises three main components: a capping group (cap), a linker region, and a zinc-binding group [220]. Modifications in the cap and linker regions aim to achieve selectivity for specific HDAC isoforms, while variations in the zinc-binding groups aim for increased potency. Pharmacophore models can be classified as structure-based, developed from protein-ligand complexes, or ligand-based, utilizing known HDAC inhibitors’ structures” were changed to “HDAC inhibitors traditionally feature a pharmacophore composed of three fundamental elements: a cap, a linker region, and a ZBG [223]. Modifications in the ZBG aim for increased potency, while variations in the cap and linker regions are designed to enhance selectivity for particular HDAC isoforms. Pharmacophore models can be classified as structure-based, which are derived from the complexes of proteins and ligands, or ligand-based, which are based on the structures of existing HDACis”; the sentences “a screening process was conducted on 300,000 compounds sourced from Asinex, National Cancer Institute (NCI) and Maybridge databases using e-pharmacophore modeling [222]. Also, a 3D chemical feature-based QSAR pharmacophore model was developed to study the interaction between benzamide MS-275 and HDAC [220]. A potent HDAC3 inhibitor was identified by Kumbhar et al., by using a combined computational screening strategy involving” were changed to “a total of 300,000 compounds sourced from the Asinex, National Cancer Institute (NCI), and Maybridge databases were screened using e-pharmacophore modeling [225]. Also, the interaction between benzamide MS-275 and HDACs was investigated by means of a 3D chemical feature-based QSAR pharmacophore model [223]. A potent HDAC3 inhibitor was identified by Kumbhar et al., by using an integrated computational screening strategy, which included”; the sentences “induced fit docking (IFD) optimized protein-ligand interactions and MD simulations with MM-GBSA calculations were employed [248]. In order to address the limitations of 3D-QSAR, researchers developed 4D-QSAR models incorporating molecular state ensemble averaging [248]” were changed to “protein–ligand interactions were refined using induced fit docking (IFD), and molecular dynamics (MDs) simulations combined with MM-GBSA calculations were also utilized to enhance the accuracy of the analysis [251]. To overcome the limitations of 3D-QSAR, researchers advanced to 4D-QSAR models that integrate molecular state ensemble averaging [251]”; the sentence “Due to the wealth of the crystal structures of HDACs, structure-based inhibitor design has become increasingly achievable” was changed to “Thanks to the abundance of crystal structures in HDACs, designing inhibitors based on their structure has become more feasible”; the sentences “These models, along with crystal structures of HDAC4 and -7, were employed to perform structure-based/molecular docking-based virtual screening of the NCI compound library, aiming at identifying potential inhibitors of Class IIa HDACs. Subsequently, these compounds underwent evaluation against HeLa nuclear Class II HDACs to uncover Class IIa-selective inhibitors [250]” were changed to “Utilizing these models and the crystal structures of HDAC4 and HDAC7, researchers conducted a virtual screening of the NCI compound library based on structure and molecular docking. This approach aimed to identify potential inhibitors of Class IIa HDACs. The identified compounds were then tested against HeLa nuclear Class II HDACs to find those with selective inhibition for Class IIa HDACs [253]”; the sentence “a compound demonstrating nanomolar activity against HDAC1, HDAC3 and HDAC6 was uncovered through a combined approach of pharmacophore modeling and structure-based virtual screening of an in-house database comprising 22,700 compounds” was changed to “the screening of an in-house compound library using a combined approach of structure-based and pharmacophore modeling led to the identification of a compound with nanomolar activity against HDAC1, HDAC3, and HDAC6”; and the sentence “The most promising candidates are subjected to MD simulations to examine the stability of ligand binding modes. For a more precise determination of ligand binding affinity” was changed to “The most promising candidates are evaluated through MD simulations to assess the stability of their ligand binding modes”.

Some corrections have been made to Section 4.4. Achieving Selectivity for Each HDAC Isoform, where the sentences “Previously, Abdizadeh et al. used combined 3D-QSAR and molecular docking approaches to design biaryl benzamides as potent HDAC1 inhibitors [312]. Cao et al. conducted a review of different modifications carried out to the cap and linker groups to achieve selectivity for HDAC3” were changed to “Previously, Abdizadeh et al. designed potent HDAC1 inhibitors by developing biaryl benzamides using a combination of 3D-QSAR and molecular docking techniques [315]. In another study, Cao et al. explored various modifications to the cap and linker regions to enhance selectivity for HDAC3”; the sentences “In the quest for HDAC6-selective inhibitors, a series of compounds incorporating 2-mercaptoquinazolinone as the cap moiety were developed. This effort focused on modifying the surface recognition group (utilizing the quinazolinone core as a cap) and linker, while preserving hydroxamic acid side chains at either the C-2 or N-3 position [218]” were changed to “A set of compounds with 2-mercaptoquinazolinone as the cap group was designed to target HDAC6 specifically. This approach focused on optimizing the surface recognition element by incorporating the quinazolinone core as the cap, along with modifying the linker structure. At the same time, it maintained the hydroxamic acid side chains at either the C-2 or N-3 positions to ensure their efficacy [221]”; the sentence “resulting in the surprising identification of aminotriazole with subnanomolar inhibitory potency and significant selectivity for Class I HDAC1 and HDAC8 [316]” was changed to “This interesting approach led to the unexpected discovery of aminotriazole, which exhibited remarkable subnanomolar potency and a notable selectivity for Class I HDACs, specifically HDAC1 and HDAC8 [319]”; and the sentences “The SBVS pointed out the ibotenic acid as a promising HDAC7 inhibitor based on its theoretical binding affinity. In vivo studies demonstrated the capability of the ibotenic acid in decreasing the cellular viability on MCF-breast cancer cells, providing an interesting example of repurposing natural products to fight cancer disease” were changed to “Using structure-based virtual screening (SBVS), ibotenic acid emerged as a potential HDAC7 inhibitor due to its predicted binding affinity. Subsequent in vivo studies revealed that ibotenic acid effectively reduced cellular viability in MCF breast cancer cells, highlighting its potential as a repurposed natural product for cancer treatment”.

With this correction, the order of some references has been adjusted accordingly. The authors state that the scientific conclusions are unaffected. This correction was approved by the Academic Editor. The original publication has also been updated.
